# Genetic Diversity and Population Structure of Maize (*Zea mays* L.) Inbred Lines in Association with Phenotypic and Grain Qualitative Traits Using SSR Genotyping

**DOI:** 10.3390/plants13060823

**Published:** 2024-03-13

**Authors:** Rumit Patel, Juned Memon, Sushil Kumar, Dipak A. Patel, Amar A. Sakure, Manish B. Patel, Arna Das, Chikkappa G. Karjagi, Swati Patel, Ujjaval Patel, Rajib Roychowdhury

**Affiliations:** 1 Department of Agricultural Biotechnology, Anand Agricultural University, Anand 388110, India; 2 Department of Genetics and Plant Breeding, B. A. College of Agriculture, Anand Agricultural University, Anand 388110, India; 3 Main Maize Research Station, Anand Agricultural University, Godhra 389001, India; 4 ICAR—Indian Institute of Maize Research, PAU Campus, Ludhiana 141004, India; 5 Department of Genetics and Plant Breeding, N. M. College of Agriculture, Navsari Agricultural University, Navsari 396450, India; 6 Department of Plant Pathology and Weed Research, Institute of Plant Protection, Agricultural Research Organization (ARO)—Volcani Center, Rishon Lezion 7505101, Israel

**Keywords:** genetic diversity, GWAS, population structure, SSR, *Zea mays*

## Abstract

Maize (*Zea mays* L.) is an important cereal and is affected by climate change. Therefore, the production of climate-smart maize is urgently needed by preserving diverse genetic backgrounds through the exploration of their genetic diversity. To achieve this, 96 maize inbred lines were used to screen for phenotypic yield-associated traits and grain quality parameters. These traits were studied across two different environments (Anand and Godhra) and polymorphic simple sequence repeat (SSR) markers were employed to investigate the genetic diversity, population structure, and trait-linked association. Genotype–environment interaction (GEI) reveals that most of the phenotypic traits were governed by the genotype itself across the environments, except for plant and ear height, which largely interact with the environment. The genotypic correlation was found to be positive and significant among protein, lysine and tryptophan content. Similarly, yield-attributing traits like ear girth, kernel rows ear^−1^, kernels row^−1^ and number of kernels ear^−1^ were strongly correlated to each other. Pair-wise genetic distance ranged from 0.0983 (1820194/T1 and 1820192/4-20) to 0.7377 (IGI-1101 and 1820168/T1). The SSRs can discriminate the maize population into three distinct groups and shortlisted two genotypes (IGI-1101 and 1820168/T1) as highly diverse lines. Out of the studied 136 SSRs, 61 were polymorphic to amplify a total of 131 alleles (2–3 per loci) with 0.46 average gene diversity. The Polymorphism Information Content (PIC) ranged from 0.24 (*umc1578*) to 0.58 (*umc2252*). Similarly, population structure analysis revealed three distinct groups with 19.79% admixture among the genotypes. Genome-wide scanning through a mixed linear model identifies the stable association of the markers *umc2038*, *umc2050* and *umc2296* with protein, *umc2296* and *umc2252* with tryptophan, and *umc1535* and *umc1303* with total soluble sugar. The obtained maize lines and SSRs can be utilized in future maize breeding programs in relation to other trait characterizations, developments, and subsequent molecular breeding performances for trait introgression into elite genotypes.

## 1. Introduction

Maize (*Zea mays* L., 2n = 20) is the most widely cultivated cereal in the world and is thus generally recognized as the ‘Queen of Cereals’ due to its high production potential. It is also renowned as a “model crop”, due to its higher genetic diversity and incorporation in basic and advanced breeding programs for genetic improvement [1]. Being the primary staple food for millions, maize is a key component of animal feed and a significant source of bioenergy. It is a high biological-yield-producing crop with remarkable photosynthetic ability due to the foliar ‘Kranz anatomy’ [2]. Maize is used in the production of biofuel, fabric starch, high-quality corn oil and alcoholic quenchers, and raw material in the pharmaceutical and cosmetic industries [2]. However, the increased human population and climate change in this present era combined with limited production resulted in the scarcity of this crop as a raw material. However, climate change associated obstacles such as yield stagnation, grain quality improvement hurdles, growing disease and pest pressures, and harsh weather circumstances have created significant quantitative and qualitative losses of maize production [3]. As a result, immediate action is essential to support genetic research to improve maize breeding efficiency. It is believed that maize can act as a climate-resilient crop in rainfed areas of tropical and subtropical climatic regions by developing specific hybrids and varieties against changing climate [4]. Maize reproduces sexually through allogamy by protandry condition, which gives the chance to create an enormous amount of genetic variability in diverse morphotype like plant height, flowering (silking and tasseling) time, maturation period, ear and kernel features, as well as diversity in its response to climate change. For many years, growers and breeders have used this variation to create cultivars with specific characteristics [5,6]. The new varieties replaced the old traditional landraces and became the cause of genetic erosion [1]. To prevent the genetic loss, continued sourcing, systematic recording, and in situ and/or ex situ storages are necessary, which also aids plant-breeding programs by providing varied maize genetic stocks. Such maize stocks can be used to create new varieties and deploy a new beneficial diversity for long-term advances in agricultural productivity and genetic advantages [7]. These efforts can be greatly aided by modern genetic improvement tools [8,9].

According to Van Inghelandt et al. [10], a basic knowledge about population structure and the genetic diversity of maize germplasm is a prerequisite for the success of a breeding program. This knowledge enables the tracking of the pattern of inheritance and the amount of polymorphism and heterozygosity [10]. Marker-assisted approaches like phenological, morphological, biochemical and molecular (DNA-based) markers are used to examine maize genetic diversity and population structure among germplasms [10]. Morphological, yield-associated components and grain quality traits of maize are frequently employed as the major breeding parameters for determining population genetic diversity. However, such traits are heavily influenced by environmental interaction. To overcome such limitations, DNA-based markers are introduced to identify cultivars and accelerate breeding programs through early selection of maize lines or segregants with the traits of interest [11,12]. A polymerase chain reaction (PCR) leveraging efficient molecular markers, particularly the simple sequence repeats (SSRs) marker, has long been pivotal in maize genetic research and breeding endeavors [13,14,15]. SSR markers are more informative than biallelic Single Nucleotide Polymorphism (SNP) since they detect multiple alleles per genomic loci [16,17]. Van Inghelandt et al. [10] reported that SSRs are 7 to 11 times more accurate than SNPs. SSRs are broadly scattered throughout the genome in both coding and non-coding regions as a small simple repeat motif [18], which makes them more effective in assessing the genetic diversity and population structure of maize landraces and improved lines [19].

The relationship between morphological data and molecular marker patterns offered important information on population structure and genetic diversity. Identification of these relations is popularly known as association mapping. Association mapping takes advantage of historical and evolutionary crossing-overs. It is based on SSR genotyping revealed linkage disequilibrium (LD) and has a greater mapping resolution than the linkage mapping approach. The statistical relation between SSRs and traits is often utilized to identify a quantitative trait loci (QTL) [3]. Instead of creating a mapping population using linkage mapping, association mapping may be carried out on natural maize populations and inbred lines. This mapping technique allows for the simultaneous evaluation of several alleles as well as the assessment of numerous recombination events, which took place across a number of generations [20].

Genetic diversity and variation analysis are essential for maize inbred lines to facilitate the development of improved varieties with desirable traits. Maize breeders rely on genetic diversity within inbred lines to select and combine alleles that confer traits such as high yield, disease resistance, abiotic stress tolerance, and nutritional quality [21]. Understanding the genetic diversity among such lines is crucial for exploiting heterosis, where hybrids exhibit superior performance compared to their parental lines. Moreover, genetic variation analysis allows breeders to identify and introgress resistance genes against diseases, pests, and environmental stresses, thereby enhancing the durability and resilience of maize cultivars. Furthermore, genetic diversity analysis aids in the improvement of nutritional quality and adaptation to changing climatic conditions, ensuring the sustainability and productivity of maize production systems [22]. In essence, genetic diversity and variation analysis serve as fundamental tools for maize breeders to develop cultivars that meet the diverse needs of farmers and consumers while addressing the challenges of modern agriculture [23].

In light of the foregoing explanation, the aim of this present study is (i) to evaluate the variability of yield-associated morphological traits and grain quality parameters, (ii) to reveal the genetic diversity and population structure of the maize inbred population using genome wide distributed SSR markers, and (iii) to validate the association between SSR markers and traits. The findings of the potential maize lines with marker-trait association will be the outcome to broadening the genetic base, creating new gene combinations and identifying markers for achieving higher genetic gain in future maize breeding programs.

## 2. Materials and Methods

### 2.1. Plant Materials and Experimental Locations

In this study, a set of 96 genetically diverse maize (*Zea mays* L.) inbred lines were used for the assessment of genetic diversity, population structure and marker-trait association using some potent yield-associated morphological traits and grain quality parameters. Among those, 36 lines belong to the sweet corn group and were obtained from the Indian Institute of Maize Research (IIMR, Ludhiana, India). The remaining 60 maize lines belong to the flour corn group and were obtained from the Main Maize Research Station (MMRS, Godhra, India). The details of studied germplasm are presented in Table S1. As we aimed to analyze the diversity and genetic variation in the maize lines, no check varieties were not included for the purpose of trait comparison. The inbred lines were field evaluated at (i) ‘Anand’, the Experimental Farm (22.54° N, 72.98° E, 45.10 m above mean sea level) of Anand Agriculture University, Gujarat, India, where its soil was sandy loam and had a poor water holding capacity with poor organic carbon content and (ii) ‘Godhra’, MMRS (22.45° N, 40° E, 119.40 m above mean sea level), Gujarat, India, where soil was sandy with medium water holding capacity and contained good organic carbon. Detailed meteorological parameters were shown in Table S2. Randomized Complete Block Design (RCBD) with two replications in each site was employed in this experiment. Each maize line was sown in a 6 m row (20 plants per row) with 30 cm intra-row and 60 cm inter-row spacing during the winter season of 2020.

### 2.2. Phenotypic Characterization

The phenological data were recorded from the sowing date and expressed in days. Silking was recorded as the date when 50% of the plants in each plot had visible styles; tasseling happened immediately preceding this growth stage, but essentially, these phases must coincide for successful pollination to occur [24]. The morphological traits like plant height (PH in cm), ear height (EH in cm), ears per plant (EPP), ear length (EL in cm), ear girth (EG in cm), number of kernel rows per ear (KRP), number of kernels per row (KPR) and number of kernels per ear (NKE) were phenotyped at maturity from five randomly selected plants of each maize line for each replication per location as per previous established by the way of Patel et al. [25]. The PH was measured the aboveground distance between ground and tassel. Ear traits (EH, EPP, EL, EG) were evaluated manually after harvesting the matured cob. After the separation of ears from the cob, yield parameters (KRP, KPR and NKE) were recorded.

### 2.3. Grain Qualititative Parameters

For the biochemical analysis of the maize grain, three randomly selected plants were harvested together, and grains were separated from cob. Then, the grains were cleaned manually, followed by sun-drying for 7 days and subsequent crushing to make corn flour. This flour was stored in a plastic bag for a further estimation of protein content (Pro%) as per Bressani et al. [26], β-carotene (Car in ppm) according to Sathya et al. [27], and lysin and tryptophan content (as µg per mL) following the protocol of Lane [28]. Total soluble sugar (TSS%) was estimated from the cobs at the dough stage following the methods of Dubious [29].

### 2.4. DNA Extraction, PCR Amplification and Gel Electrophoresis

The total genomic DNA of each field-evaluated maize genotype was isolated from the 15-days-old leaves as per the protocol described by Doyle [30]. The DNA quality was checked through nanodrop (ND-1000, Thermo Fisher Scientific, Waltham, MA, USA) and 1% (*w*/*v*) agarose gel electrophoresis. A total of 136 SSR markers distributed across the maize genome were randomly selected from the MaizeGBD database (https://maizegdb.org/data_center/ssrid (accessed on 1 June 2023)) and employed to evaluate genetic variations and association analysis (Table S3). PCR amplification was carried out using Emerald 2× PCR master mix (Takara, Tokyo, Japan). The PCR reaction consisted of 5 μL of PCR master mix, 2 μL of 50 ng DNA, 1 μL of 10 pmole forward and reverse primers in a total volume reaction of 10 μL. The volume was adjusted with nuclease-free water. The thermal cycling conditions were as follows: an initial denaturation at 94 °C for 5 min, followed by 35 cycles of denaturation at 94 °C for 45 s, annealing at ΔT °C (primer specific) for 45 s and extension at 72 °C for 45 s; one cycle of final extension was performed at 72 °C for 7 min. The PCR reactions were conducted in Applied Biosystems^®^ Veriti^®^ 96-Well Thermal Cycler (Thermo Fisher Scientific, Waltham, MA, USA). A 3% agarose gel was used to resolve PCR products with a 100 bp DNA ladder. Allele scoring was performed using the software AlphaEaseFC 4.0 and allele size was determined for downstream data processing and statistical genetic analysis.

### 2.5. Statistical Analysis

The phenotypic field data and biochemical estimations were examined through combined analysis of variance (ANOVA) by assuming genotypic effects as fixed and environment effects as random. To determine the association between traits, the analysis of correlation was performed using experimental means for each variety from each location. The descriptive statistical analysis (mean values, correlation, regression, *t*-test) was carried out using JMP software v16.0 [31]. The genotypic correlation coefficient was calculated for both the environments using R v4.2.3. package “variability” [32].

### 2.6. Analysis of Genetic Diversity, Population Structure and Marker-Trait Association

The PowerMarker 3.25 program was used to calculate the allele number, allelic frequency, gene diversity (GD), and polymorphic information content (PIC) for each used SSR as suggested by Lui [33]. The genetic dissimilarities were estimated for each pair of accessions using the dissimilarity index. The inter-genotype genetic distances were generated using DARwin 6.0 with the Neighbor-Joining (NJ) method and the dendrogram was created. To infer the population structure, the Bayesian clustering approach was used through STRUCTURE 2.3.4 software as advised by Pritchard et al. [34]. For population structure, 5 runs for each ‘K’ value from 2 to 10 were performed with 500,000 burn-in and 500,000 MCMC (Markov Chain Monte Carlo). The structure harvester online program (http://taylor0.biology.ucla.edu (accessed on 23 September 2023)) was used to obtain the final ‘K’ value based on the lowest negative number of Ln (the log-likelihood of the data) and the lowest standard deviation was found during analysis, according to the protocol of Earl and VonHoldt [35]. The association of the trait of interest with each SSR allelic locus was tested through a Mixed Linear Model (MLM) using TASSEL 3.0 software [36]. The expected value under the null hypothesis of no relationship with the trait was displayed against the negative logarithms of the *p*-values from the models used in the GWAS in this graph. The quantile–quantile (QQ) plot is an important tool for evaluating the efficiency of the model employed in GWAS for structuring the population and recognizing familial relatedness. The QQ plot was derived from the *p*-value of MLM through the R package v4.2.3. “CMplot” [37]. The Manhattan scatter plot was developed through the R package “qqman” to summarize GWAS results according to the procedure explained by Turner [38]. The Y-axis is the negative logarithm of the *p*-value derived from the GWAS model (particularly, from the F-test for testing H_0_: No connection between the SSR and trait) and the X-axis is the genomic location of each SSR. Large peaks in the Manhattan plot indicated a substantial relationship between the characteristic and the adjacent genetic region.

## 3. Results

### 3.1. Phenotypic Evaluation and Genotypic Mean Performance in Two Environments

The combined analysis of variance indicated that all the characters were found to be significant for the genotype, implying the presence of significant variability for all characters of the panel under study (Table 1). This is the prerequisite for a successful breeding program. It also showed a significant effect of the environment which indicated a significant influence of environment on the expression of the particular traits. According to Table 1, plant height (37.92%) and lowest ear placement height (48.57%) had the highest influence on the environment indicating that differences in a microenvironment like soil fertility play a significant role in the expression of these characteristics.

**Figure 1:**
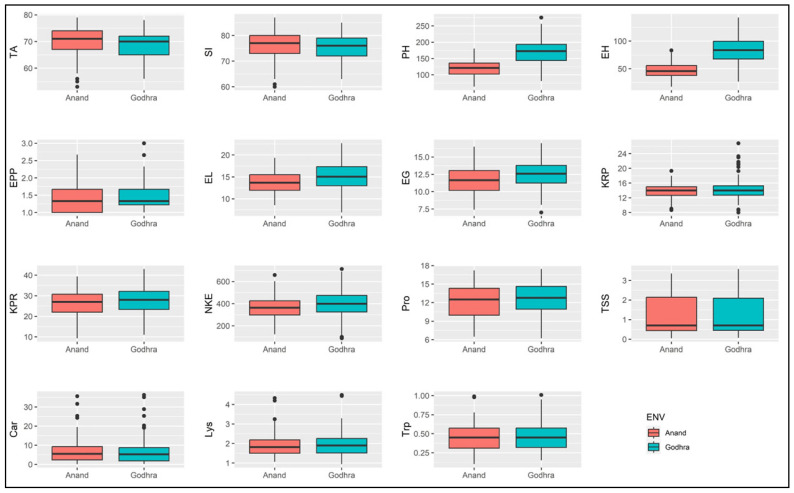
Box plots showing mean performance of the studied traits across two test sites during 2020. TA: days to 50% tasseling, SI: days to 50% silking, PH: plant height, EH: ear height, EPP: ears per plant, EL: ear length, EG: ear girth, KRP: kernel rows per ear, KPR: kernels per row, NKE: number of kernels per ear, Pro: protein content, TSS: total soluble sugar, Car: carotene content, Lys: lysine content, Trp: tryptophan content.

Similarly, ears per plant showed the highest influence on the interaction of genotype, with the environment or total variance towards overall variability (54.83%) followed by kernel rows per ear (52.70%) and ear girth (44.91%). In the rest of the characteristics, genotype shared maximum variation followed by GEI effect across all locations (Table 1; for the mean differences see Table S4). Figure 1 depicted the mean over the environment and mean of the site, respectively. The overall mean of the studied characters was 69.38 (TA), 75.73 (SI), 144.12 (PH), 65.45 (EH), 1.46 (EPP), 14.38 (EL), 12.08 (EG), 13.99 (KRP), 27.22 (KPR), 381.86 (NKE), 12.33 (Pro), 1.24 (TSS), 6.46 (Car), 1.94 (Lys) and 0.46 (Trp). According to the boxplot, most of the characters showed a similar performance across the environment except for the plant height, ear height, ear length and ear girth (Figure 1). This result was also confirmed using the combined ANOVA.

### 3.2. Genotypic Correlation between the Studied Traits

The genotypic correlation among 14 characters of 96 genotypes evaluated at the Anand (lower side of the diagonal value) and Godhra (upper side of the diagonal value) locations are shown in Table 2. Days to 50% tasseling and silking were negatively associated with the plant height and ear height at both locations. This indicates the selection of the early flowering genotype can be carried out through selecting a dwarf line, and this could lead to early maturity. Similarly, yield-attributing traits like ear length were highly correlated with the number of kernels per row, ear girth and number of kernels per ear at both locations (Table 2). Likewise, in this observation, all yield-attributing characters were also associated positively with each other at both sites.

### 3.3. Genetic Diversity among Maize Genotypes

The genetic diversity of the 96 genotypes of maize was evaluated using SSR markers. In the current study, 136 SSR markers were screened for polymorphism but among them 61 markers showed effective polymorphism (Figure S1). A total number of 131 alleles were perceived ranging from 2 alleles per loci to 3 alleles per loci with an average of 2.14 alleles per loci. The molecular weight of the markers ranged from 100 bp (*umc2298* and *umc2226*) to 930 bp (*umc1480*) (Table 3). The major allele frequency ranged from 0.41 (*umc2252*) to 0.83 (*umc2282*) with an average of 0.63. Also, the average gene diversity was recorded 0.46 with the range of 0.28 (*umc1578*) to 0.65 (*umc2252*). Heterozygosity varied widely from 0.00 to 0.82 (*umc2252*) with an average of 0.04. The informativeness in form of the strength as a marker for the polymorphism-based genotypic discrimination ability is measured using the Polymorphism Information Content (PIC) value. The PIC ranged from 0.24 (*umc1578*) to 0.58 (*umc2252*) with an average of 0.36 (Table 3). In the present study, *umc2252* (0.58), *umc1060* (0.51) and *umc1552* (0.54) were highly informative, while the rest of the markers were moderately informative (Table 3).

### 3.4. Inter-Genotypic Genetic Relationship and Clustering

**Figure 2:**
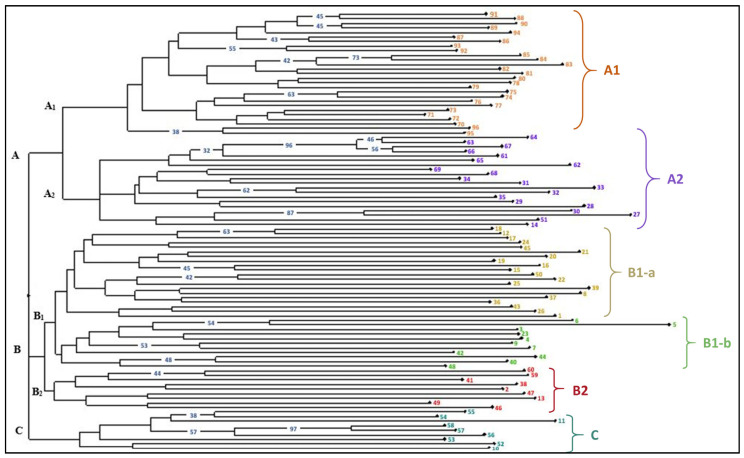
Dendrogram showing the relationship among 96 maize genotypes using molecular marker data (name of genotypes corresponds to the numbers are mentioned in Table S1).

A total of 96 maize genotypes were clustered into three foremost clusters A, B and C with 47, 40 and 9 genotypic entries, respectively, based on the pair-wise comparison values of Nei’s genetic distance (Figure 2). The maximum number of maize lines grouped in cluster A showing those lines are genetically similar. The main cluster A was further separated into two sub clusters A1 (27 lines) and A2 (20 lines). All 27 lines grouped in sub-cluster A1 comprised only sweet corn type. Sub-cluster A2 had 20 lines, which comprised flour corn as well as sweet corn type. Cluster B was further separated into two sub clusters B1 (31 lines) and B2 (9 lines). The maximum number of accessions were clustered in sub-cluster B1 belonging to flour corn type of inbreds, showing high genetic resemblance among genotypes of this group. Pair-wise genetic distance values among 96 maize lines ranged from 0.0983 (1820194/T1 and 1820192/4-20) to 0.7377 (IGI-1101 and 1820168/T1) (Table S5).

### 3.5. Population Structure Analysis

**Figure 3:**
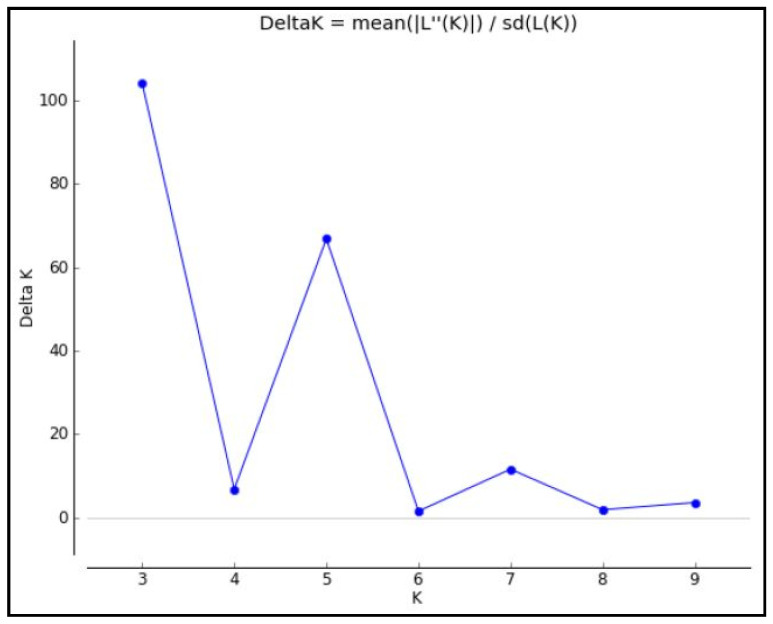
Estimation of hypothetical sub-populations using ΔK-values.

**Figure 4:**
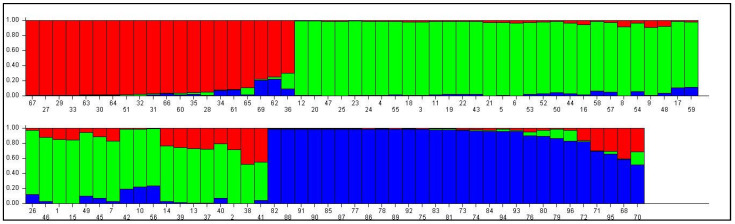
Model-based population structure plot for each genotype with K = 3 in STRUCTURE software using 61 polymorphic SSRs. Color codes are as follows: sub-population I red, sub-population II green and sub-population III blue. The single vertical line represents an individual genotype and different segments of each vertical line show extent of admixture in an individual genotype.

STRUCTURE 2.3.4 was used to minimize type I errors during the genome-wide association study. In this study, a total of 61 polymorphic SSR markers were accessed to determine the population structure. Admixture model-based simulations were performed by varying the value of K from 2 to 10 for each K with five repetitions. A sharp pick with the maximum value of delta K was obtained at K = 3 with the lowest standard deviation (Figure 3 and Figure 4, Table S6), confirming the three sub-populations in the present mapping panel using the Evanno method (Figure 3).

Three (K = 3) optimum numbers of sub-populations were estimated which potentially explains the population structure of the accessions (Figure 4). Using this approach, 96 genotypes of maize were assigned to three sub-populations representing the 17.70% with 17 genotypes (sub-population I), 37.50% with 36 genotypes (sub-population II) and 25.00% with 24 genotypes (sub-population III) (Table 4). But based on the membership probability threshold of 0.80, 19 inbred lines were assigned to an admixed group with the representation of 19.79% of the whole panel (Figure 4, Table S7). Furthermore, the net nucleotide distance among the sub-populations was computed using point estimates of P (Table 4). This distance showed that sub-population I and III showed the maximum net nucleotide distance while sub-population I and II showed the lowest distance. Similarly, sub-population II and III showed a moderate distance. There was no observed difference found in the expected heterozygosity in the populations. Sub-population III was less heterozygous with an expected heterozygosity value of 0.3111, followed by subpopulation I with a value of 0.3729 (Table 4). The highest expected heterozygosity was shown by sub-population II. However, sub-population II reported less diversity with the lowest Fst value (0.1368), while subpopulation III reported a maximum Fst value (0.3591) with great diversity among sub-populations. But in the present study, sub-population II implies the genotypes from cluster B and C of Nei’s genetic distance-based dendrogram which is genetically more diverse (Figure 2).

### 3.6. Trait-Marker Association

**Figure 5:**
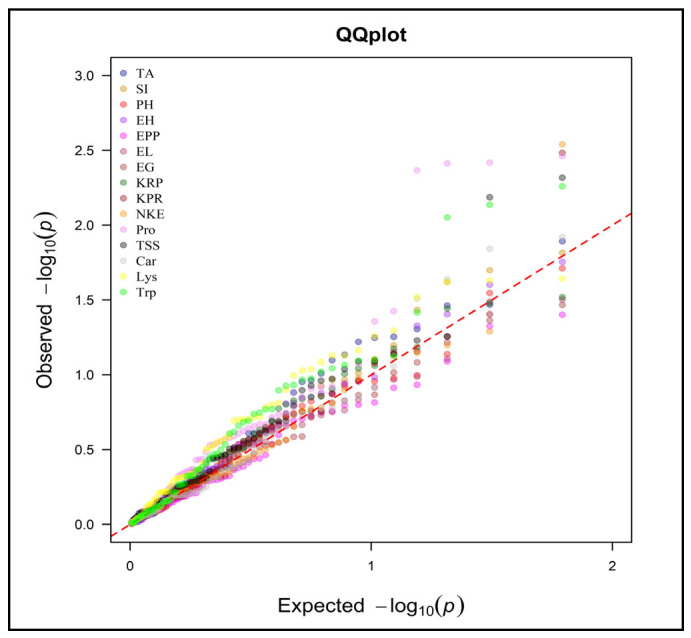
QQ plot for MLM of the studied traits in Anand environment.

**Figure 6:**
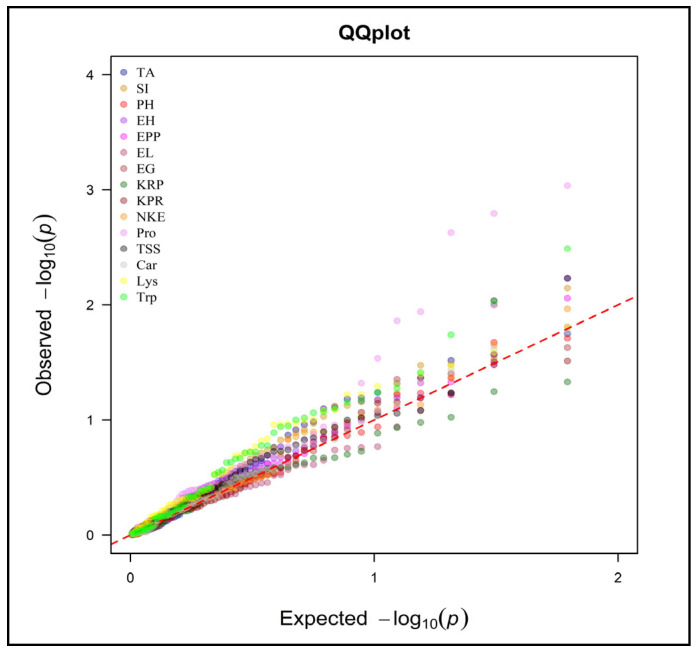
QQ plot for MLM of the studied traits in Godhra environment.

A combined model is used for mixed model frameworks (Q + K) for marker-trait associations. The data obtained from population structure analysis (Q-matrix) and kinship (K-matrix) are used for the mixed model framework (Q + K) for marker-trait associations. In this study, the marker-trait association was performed for all recorded traits at both the locations and all polymorphic markers using the Q + K MLM approach through TASSEL. Significant marker-trait association was identified by using the Bonferroni threshold [39]. By performing genome scanning through the Q + K MLM approach, marker-trait associations were found for both locations. QQ plots were used to visualize the result of the Q + MLM approach (Figure 5, Figure 6 and Figures S2 and S3). This observed the significant deviation from the diagonal line due to population structure and familial relatedness for the traits. According to the QQ plot for the Anand environment, the number of kernels per row, number of kernels per ear, protein content, total soluble sugar content and tryptophan content showed significant deviation (Figure 5 and Figure S2).

**Figure 7:**
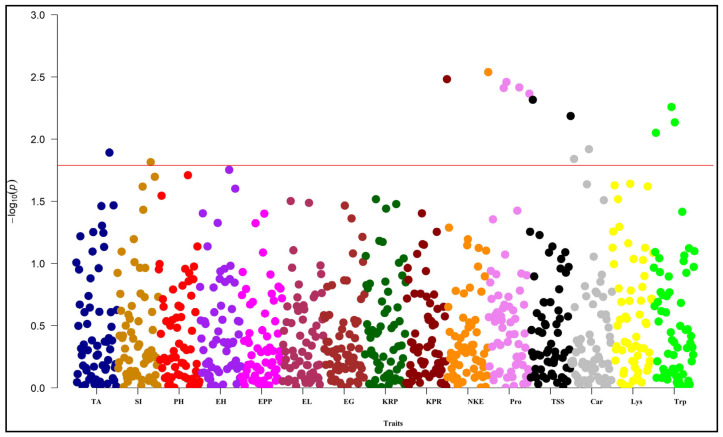
Manhattan plot for MLM of the studied traits under Anand environment. The horizontal red line indicates the threshold value. TA: days to 50% tasseling, SI: days to 50% silking, PH: plant height, EH: ear height, EPP: ears per plant, EL: ear length, EG: ear girth, KRP: kernel rows per ear, KPR: kernels per row, NKE: number of kernels per ear, Pro: protein content, TSS: total soluble sugar, Car: carotene content, Lys: lysine content, Trp: tryptophan content.

**Figure 8:**
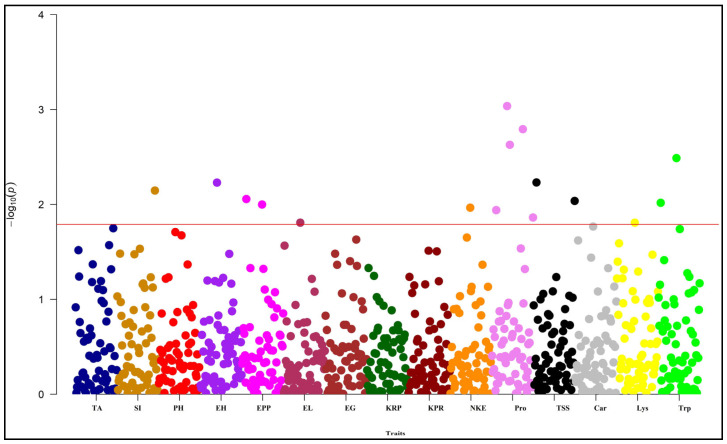
Manhattan plot for MLM of the studied traits under Godhra environment. The horizontal red line indicates the threshold value. TA: days to 50% tasseling, SI: days to 50% silking, PH: plant height, EH: ear height, EPP: ears per plant, EL: ear length, EG: ear girth, KRP: kernel rows per ear, KPR: kernels per row, NKE: number of kernels per ear, Pro: protein content, TSS: total soluble sugar, Car: carotene content, Lys: lysine content, Trp: tryptophan content.

Similarly, all these characters also surpassed the threshold in the Manhattan plot (Figure 7 and Figure 8). According to the QQ plot and Manhattan plot, 10 markers showed association with the four yields attributing and the 4-grain biochemical traits with an average of 25.46% phenotypic variance in the Anand location (Figure 7). Table 5 indicates that *umc2038* is strongly associated with the protein content, with 13.09% phenotypic variance, followed by *umc2050* with 12.74% phenotypic variance. *umc2296* and *umc2136* showed a strong association with tryptophan content. Yield-attributing traits like the number of kernels per row and number of kernels per ear were strongly associated with the umc1303 with 9.57% and 9.85% phenotypic variance, respectively (Figure 7 and Figure 8).

Similarly, the QQ plot (Figure 6 and Figure S3) and Manhattan plot (Figure 8) for the Godhra location indicated the significance of markers for the days up to 50% silking, ear height, ears per plant, protein content, total soluble sugar content, lysine and tryptophan content. In this location, yield-attributing traits like the number of kernels per ear, ear length and ears per plant were associated with *umc1941*, *umc2384* and *umc2380* (Table 5). Additionally, protein content was strongly associated with the *umc2038* with 17.28% phenotypic variance. This was followed by the *umc2050* and *umc2296* with 15.09% and 13.83% phenotypic variance, respectively (Figure 7 and Figure 8, Table 5). An *umc2296* was associated with lysine and tryptophan content with 9.18 and 12.85% phenotypic variance, respectively. In this study, *umc2038*, *umc2050* and *umc2296* were found to be strongly associated with protein content in both environments, indicating a stable association. Similarly, *umc2296* and umc2252 showed a significant and strong association with tryptophan content at both locations. The total soluble sugars also established a significant association with *umc1535* and *umc1303* at both locations (Table 5).

## 4. Discussion

The present study reveals the significant diversity of maize inbred lines for yield-associated morphological and grain biochemical traits supported using the combined ANOVA (Table 1). Within the genotype, the very least significant variation has been observed. As the studied population are inbred lines, it was quite expected due to their homozygous genetic constituents. Those lines were maintained through continuous selfing in the field condition by restricting the open pollination through bagging the tassel and cobs. During the season, no such morphological visible variation was observed. It is very prominent from Table 1 that the highest variation comes from GEI, which is in fact the consequence of genotype–environment interaction (G × E). Similar trait diversity was previously reported in other maize lines [25,40]. Patel et al. [41] showed the environment shared the least variation but its significance indicated the variability in the test sites. In the present study, grain quality parameters shared maximum variability through genotypic effects, proving its stability across the two environments in Anand and Godhra.

In our study, genotypic correlation analysis expresses the relatedness among the traits from their genetic counterparts from the total variance. The significant and negative correlation among days up to 50% tasseling and silking with plant height helps to select early maturing genotypes [25]. Patel et al. [25] recorded a significant and positive correlation between the ear length to number of kernels per row (0.64) and the number of kernels per ear (0.54). Patel et al. [42] also recorded the same trend with ear length to number of kernels per row (0.67) and number of kernels per ear (0.47). Protein content is significantly associated with the lysine and tryptophan content in a positive direction at both locations. This significant correlation is observed because protein is an important structural composition of maize grain while lysine and tryptophan are contributing amino acids in the development of quality protein maize. Diversity based on phenotypic performance gives the idea about the potential of genotype, but it is highly vulnerable to environmental conditions. Changing the environment leads to the biased estimation of population diversity because of the G × E interaction.

To overcome the environment and stage specificity effect, molecular markers are introduced in the plant breeding research. SSR-based genetic diversity shows the markers’ efficiency of polymorphism is less than 50%. However, the amplified alleles are in a moderate range (2–3 alleles per locus). This is why total gene diversity and PIC values are not much higher than moderate. Based on these parameters, three SSRs (*umc2252*, *umc1060* and *umc1552*) are found to be more informative to discriminate the maize lines. All these three SSRs amplify three alleles per locus and are positioned in chr. 2 and 5. Similarly, Sa et al. [43] reported 3 to 15 alleles per loci with an average of 7.6 during the genetic diversity analysis of Canadian maize inbred lines. Similarly, Adu et al. [44] reported 4 to 17 alleles per loci with an average of 9.60 in the maize populations. The difference in allele numbers in the present study and the previous study is because of the difference in the methodology of polymorphism detection and plant genetic material used in the study [44]. SSR markers showed 0.46 average gene diversity with a range of 0.28 to 0.65. This result is in accordance with the Sa et al. [43] in maize. However, Park et al. [45] noticed that gene diversity values varied from 0.20 to 0.92 with an average of 0.65, which was higher than the present study. Many of the markers show a unique inbreeding coefficient which indicates less or no heterozygosity. Because maize is a highly cross-pollinated crop, in the present study, inbreds were used and were maintained through continuous inbreeding. This process prevents the gene flow between two populations that may prevent blending of two gene pools thereby decreasing the genetic variation and increasing the homozygosity. However, the true value of markers can be predicted using their PIC value. As per the Botstein et al. [13], the PIC value of a marker is classified into three main categories viz. high, moderate and less informative. The highly informative SSRs have a PIC value greater than 0.5, the moderately informative have a PIC within the range of 0.25–0.5 and the slightly or less informative marker have a PIC below 0.25. In the present study, *umc2252*, *umc1060* and *umc1552* were found to be highly informative and the rest of the markers were moderately informative. A high to moderate PIC value indicated the multi-allelic behavior of the SSR markers. Adu et al. [44] concluded that the high magnitude of the polymorphism of SSR markers supports its application in various genetic studies like genetic diversity, QTL mapping and Genome Wide Association Studies (GWAS). Wasala et al. [46] revealed heterozygosity values ranging from 0.13 to 0.75 with an average of 0.63, and polymorphic information content values ranging from 0.20 to 0.90 with a mean of 0.60, which were comparable to the current findings. The SSR-based genotyping enabled the grouping of the studied maize lines into three foremost clusters, which can easily classify the maize lines into sweet corn and flour corn types. The pair-wise comparison based on Nei’s genetic distance ranged to high, from 0.0983 (1820194/T1 and 1820192/4-20) to 0.7377 (IGI-1101 and 1820168/T1), which is clearly indicated in the population structure (Figure 4). The inter-genotype genetic relationship indicated that the genotype IGI-1101 and 1820168/T1 were extremely diverged at the genomic level and can be utilized to create biparental mapping populations as well as in a maize improvement program to broaden the genetic make-up of various maize genotypes. This is in much accordance with the findings of Ganguniya et al. 2023 on another cereal relative pearl millet [47]. Similar results were also inferred by Saiyad et al. 2018 [48] in fodder maize with a maximum Nei’s genetic distance of 0.16 between ‘IC-130791’ and ‘IC-130759’, with the minimum distance of 0.75 found between ‘Origin Mexico-6386’ and ‘Mexico genotype-3970’.

Marker information and cluster analysis help to select a genotype to broaden the genetic base of a crop, but it cannot offer an idea about how genetic variation in population is distributed among and within the population. Population structure analysis is used to infer information regarding genetic variations in and among populations. It is a prerequisite for GWAS. Without the population structure analysis, population stratification underwrites to false positive results in the GWAS [20]. Sometimes pseudo-associations are also found because the population was structured according to the geographical origin of the genotype. Therefore, to avoid the high rate of type I error, a suitable and effective method is implemented to control the effect of population structure during association analysis using STRUCTURE [20]. Population structure analysis divided 96 genotypes into three subpopulations with 17.70% (subpopulation I), 37.50% (subpopulation II) and 25.00% (subpopulation III) representation. Among them, 19.79% of genotypes were assigned to admixed groups based on the membership probability threshold value of 0.80. Admixtures in the populations can be ascribed mostly owing to the allogamous nature of crop and gene flow from the adjacent genotype. The knowledge gained from population structure and ancestry would help with germplasm management and conservation strategies, as well as the choice of suitable parents in maize breeding programs. Sub-population I and II showed minimum net nucleotide distance indicating minimal changes in allele frequencies between these sub-populations and vice versa [44]. The study revealed that sub-population II was more genetically diverse and more heterozygous compared to other sub-populations as per Holsinger et al. [49] based on expected heterozygosity. The fixation index (Fst) is a measure of population differentiation due to genetic structure which ranges from 0 to 1. According to Holsinger et al. [49], a zero value implies that the population can complete random matting. Fst equals one, implying that all genetic variation is explained through the population structure and that the two populations do not differ in their genetic composition. In this study, the fixation index found 0.35 for sub-population III, which was the highest, while 0.13 was found for sub-population II, which was the lowest. A similar conclusion was drawn by Adu et al. [44] during the genetic characterization and population structure of maize populations using SSR markers. They reported two subpopulations with a moderate Fst value of 0.18 and 0.013. They also reported less expected heterozygosity of 0.44 between populations.

Genetic diversity is the prerequisite for the beginning of any breeding program. Higher diversity in any breeding population leads to the discovery of new effective loci and improves the understanding of the genetic architecture and behavior of different loci in the population. The result of the population structure analysis supported the performance of GWAS. It helps to discover numerous genetic variations which are connected to complex characteristics. Compared to linkage mapping, association analysis gives more options to plant breeders for choosing a genotype for crop improvement [43]. In this study, none of the marker-trait associations were supported by the previous study because of the different markers and traits used in the study.

## 5. Conclusions

This study reported trait diversity, genetic structure and marker-trait association among 96 maize genotypes, through which we are able to identify potential lines for the improved agronomic trait with the best grain biochemical parameters. The multiple allelism of the SSR marker in this study proved its efficiency over the SNP marker and identified two highly diverse genotypes IGI-1101 and 1820168/T1 at the genomic level. This can be further utilized to broaden the genetic base of the maize populations. Population structure analysis divides these lines into three distinct groups, with additional admixed groups with moderate Fst values indicating the free flow of genes among groups. This revealed the absence of population structure, which opens the doors for GWAS analysis. Marker trait association through the MLM approach identified that *umc2038*, *umc2050* and *umc2296* were strongly associated with the protein content; markers *umc2296* and *umc2252* were associated with tryptophan content and markers *umc1535* and *umc1303* were strongly associated with total soluble sugar at both the locations. Surprisingly, protein, lysine and tryptophan contents are highly correlated at the genotypic level across the environment, helping to improve multiple characters simultaneously through marker-assisted breeding because as per the analysis of variance, these traits also had a good buffering capacity across the wide range of environments as these biochemical traits were potentially expressed through the genotype.

## Figures and Tables

**Table 1:** Combined analysis of variance (ANOVA) for the studied phenotypic and grain quality traits under Anand and Godhra environments.

Source of Variation	Environment (df = 1)		Genotype (df = 95)		GEI (df = 95)		Residual (df = 190)	CV (%)	Mean over Environment
Trait	Mean Squares	(G + E + GEI) %	Mean Squares	(G + E + GEI)%	Mean Squares	(G + E + GEI)%	Mean Squares		
TA	301.04 **	3.19	92.24 **	92.88	3.90	3.93	4.73	3.14	69.38
SI	229.71 **	2.58	86.62 **	92.54	4.56	4.87	5.07	2.97	75.73
PH	219,940.00 **	37.92	2038.00 **	33.38	1752.00 **	28.70	141.00	8.24	144.12
EH	134,578.50 **	48.57	777.90 **	26.67	721.90 **	24.75	50.80	10.89	65.45
EPP	0.35 **	0.76	0.22 **	44.40	0.27 **	54.83	0.07	17.55	1.46
EL	213.68 **	7.62	17.03 **	57.69	10.24 **	34.69	1.25	7.77	14.38
EG	55.88 **	4.87	6.06 **	50.21	5.42 **	44.91	1.19	9.01	12.08
KRP	24.82 **	1.50	7.96 **	45.80	9.16 **	52.70	1.20	7.82	13.99
KPR	226.37 **	1.71	83.67 **	60.01	53.38 **	38.28	3.94	7.30	27.22
NKE	108,631.00 **	2.62	24,231.00 **	55.51	18,280.00 **	41.87	1793.00	11.09	381.86
Pro	11.31 **	0.48	23.78 **	95.91	0.90 **	3.61	0.64	6.49	12.33
TSS	0.01 **	0.00	3.34 **	99.29	0.02 **	0.71	0.01	6.50	1.24
Car	7.55 **	0.06	132.19 **	97.82	2.86 **	2.12	0.84	14.17	6.46
Lys	0.08 **	0.06	1.42 **	98.81	0.02 **	1.13	0.01	5.50	1.94
Trp	0.01 **	0.05	0.12 **	98.17	0.002 **	1.78	0.001	5.96	0.46

** Significant at *p* < 0.01; df: degrees of freedom, TA: days to 50% tasseling, SI: days to 50% silking, PH: plant height, EH: ear height, EPP: ears per plant, EL: ear length, EG: ear girth, KRP: kernel rows per ear, KPR: kernels per row, NKE: number of kernels per ear, Pro: protein content, TSS: total soluble sugar, Car: carotene content, Lys: lysine content, Trp: tryptophan content, G: genotype sum of square, E: environment sum of square, GEI: genotype–environment interaction sum of square.

**Table 2:** Correlation coefficient of the studied phenotypic and grain quality traits for the evaluated maize lines across environments: Anand (lower side of the diagonal values in Italics) and Godhra (upper side of the diagonal value).

	TA	SI	PH	EH	EPP	EL	KPR	EG	KRP	NKE	Pro	TSS	Car	Lys	Trp
TA	**1.00**	0.99 **	−0.09	0.04	−0.24 *	−0.01	−0.08	−0.21 *	−0.05	−0.08	−0.07	0.06	0.20 *	−0.11	−0.12
SI	0.98 **	**1.00**	−0.09	0.04	−0.26 **	−0.02	−0.08	−0.20 *	−0.04	−0.08	−0.05	0.06	0.19	−0.10	−0.11
PH	−0.18	−0.22 *	**1.00**	0.87 **	0.26 **	0.63 **	0.41 **	0.55 **	0.04	0.34 **	−0.16	−0.13	0.02	−0.13	−0.15
EH	−0.12	−0.13	0.76 **	**1.00**	0.21 *	0.56 **	0.37 **	0.48 **	0.01	0.29 **	−0.20 *	−0.15	0.01	−0.21 *	−0.22 *
EPP	−0.09	−0.09	0.00	−0.12	**1.00**	0.07	−0.01	−0.02	−0.05	−0.05	−0.31 **	−0.39 **	−0.16	−0.06	−0.10
EL	−0.10	−0.09	0.34 **	0.29 **	−0.10	**1.00**	0.73 **	0.62 **	0.00	0.57 **	0.02	−0.09	−0.01	0.06	0.11
KPR	0.02	0.04	0.38 **	0.29 **	−0.11	0.66 **	**1.00**	0.50 **	0.09	0.82 **	0.04	0.04	0.16	0.04	0.03
EG	0.29 **	0.31 **	0.07	0.19	0.07	0.24 *	0.35 **	**1.00**	0.35 **	0.58 **	0.04	0.01	−0.08	−0.04	0.04
KRP	0.29 **	0.30 **	0.07	0.03	0.05	−0.10	0.28 **	0.61 **	**1.00**	0.63 **	0.11	0.02	−0.02	−0.02	0.04
NKE	0.11	0.12	0.32 **	0.24 *	−0.09	0.52 **	0.93 **	0.51 **	0.58 **	**1.00**	0.10	0.02	0.10	0.01	0.04
Pro	0.02	0.01	−0.15	−0.04	−0.15	0.11	0.29 **	0.14	0.08	0.27 **	**1.00**	0.60 **	0.17	0.35 **	0.51 **
TSS	0.19	0.21 *	−0.23 *	−0.24 *	0.00	−0.02	0.17	0.35 **	0.33 **	0.26 **	0.70 **	**1.00**	0.33 **	0.39 **	0.39 **
Car	0.23 *	0.26 **	−0.18	−0.33 **	0.18	−0.04	0.18	0.15	0.18	0.21 *	0.12	0.24 *	**1.00**	0.02	−0.03
Lys	−0.01	−0.01	−0.02	−0.05	−0.17	0.08	0.21 *	0.01	−0.11	0.12	0.33 **	0.37 **	0.01	**1.00**	0.91 **
Trp	−0.04	−0.03	−0.06	−0.05	−0.13	0.06	0.21 *	−0.06	−0.14	0.12	0.51 **	0.44 **	−0.09	0.84 **	**1.00**

*, ** Significant at *p* < 0.05 and *p* < 0.01, respectively. TA: days to 50% tasseling, SI: days to 50% silking, PH: plant height, EH: ear height, EPP: ears per plant, EL: ear length, EG: ear girth, KRP: kernel rows per ear, KPR: kernels per row, NKE: number of kernels per ear, Pro: protein content, TSS: total soluble sugar, Car: carotene content, Lys: lysine content, Trp: tryptophan content.

**Table 3:** Genotyping data using SSR markers across the studied maize lines.

SSR Markers	Chromosome Number	Amplicon Size (bp)	Number of Alleles	Major Allele Frequency	Gene Diversity	Heterozygosity	Polymorphism Information Content	Inbreeding Coefficient
*umc2223*	1	166–180	2	0.64	0.46	0.00	0.35	1.00
*umc1122*	1	150–170	2	0.79	0.33	0.00	0.28	1.00
*umc2151*	1	125–140	2	0.55	0.49	0.00	0.37	1.00
*umc2083*	1	122–143	2	0.55	0.49	0.13	0.37	0.74
*umc1446*	1	156–168	2	0.64	0.46	0.00	0.36	1.00
*umc1558*	1	123–151	2	0.70	0.42	0.02	0.33	0.94
*umc1353*	1	232–260	2	0.60	0.48	0.00	0.37	1.00
*umc2204*	1	149–160	2	0.64	0.46	0.00	0.35	1.00
*umc2226*	1	100–115	2	0.51	0.50	0.07	0.37	0.87
*umc2189*	1	163–175	2	0.58	0.49	0.00	0.37	1.00
*umc1452*	1	118–124	2	0.64	0.46	0.00	0.36	1.00
*umc2240*	1	160–165	2	0.53	0.50	0.00	0.37	1.00
*umc1756*	2	164–170	3	0.78	0.35	0.02	0.29	0.94
*umc1552*	2	130–170	3	0.54	0.60	0.04	0.54	0.93
*umc1262*	2	157–170	2	0.65	0.46	0.00	0.35	1.00
*umc2252*	2	112–145	3	0.41	0.65	0.82	0.58	−0.25
*umc1535*	2	155–170	2	0.53	0.50	0.00	0.37	1.00
*umc2380*	2	121–132	3	0.63	0.53	0.02	0.48	0.96
*umc2129*	2	100–166	3	0.53	0.53	0.08	0.43	0.84
*umc1256*	2	173–200	2	0.51	0.50	0.00	0.37	1.00
*umc2220*	2	114–127	2	0.67	0.44	0.00	0.35	1.00
*umc1746*	3	105–112	2	0.73	0.40	0.07	0.32	0.81
*umc1641*	3	111–134	2	0.63	0.47	0.09	0.36	0.80
*umc1010*	3	116–130	2	0.68	0.44	0.04	0.34	0.90
*umc1813*	3	123–134	2	0.69	0.43	0.01	0.34	0.98
*umc1136*	3	145–168	2	0.60	0.48	0.04	0.36	0.91
*umc1489*	3	128–146	2	0.54	0.50	0.07	0.37	0.85
*umc1690*	3	110–116	2	0.52	0.50	0.13	0.37	0.74
*umc2258*	3	151–160	2	0.65	0.46	0.01	0.35	0.98
*umc2101*	3	157–180	2	0.64	0.46	0.10	0.36	0.78
*umc2103*	3	156–175	2	0.59	0.48	0.02	0.37	0.96
*umc1578*	3	168–185	2	0.83	0.28	0.00	0.24	1.00
*umc2050*	3	131–140	2	0.70	0.42	0.06	0.33	0.85
*umc2104*	3	126–130	2	0.68	0.43	0.00	0.34	1.00
*phi193225*	3	145–153	2	0.59	0.48	0.02	0.37	0.96
*umc1757*	4	147–165	2	0.70	0.42	0.04	0.33	0.90
*umc1294*	4	150–175	2	0.77	0.35	0.01	0.29	0.97
*umc2282*	4	335–420	2	0.83	0.29	0.07	0.25	0.75
*umc2038*	4	150–160	2	0.51	0.50	0.06	0.37	0.89
*dupssr34*	4	177–233	2	0.70	0.42	0.16	0.33	0.63
*umc2384*	4	100–117	3	0.56	0.54	0.05	0.45	0.90
*umc1228*	4	141–156	2	0.60	0.48	0.00	0.36	1.00
*umc2137*	4	138–156	2	0.58	0.49	0.00	0.37	1.00
*umc1303*	4	113–120	2	0.65	0.46	0.00	0.35	1.00
*umc2388*	5	257–400	2	0.78	0.35	0.14	0.29	0.60
*umc2296*	5	133–155	2	0.67	0.44	0.13	0.34	0.71
*umc1060*	5	167–246	3	0.45	0.60	0.00	0.51	1.00
*umc2201*	5	190–210	2	0.74	0.39	0.01	0.31	0.97
*umc1941*	5	106–131	3	0.56	0.55	0.00	0.46	1.00
*phi087*	5	116–129	2	0.76	0.37	0.02	0.30	0.94
*umc2136*	5	149–181	2	0.66	0.45	0.04	0.35	0.91
*umc2295*	5	129–136	2	0.56	0.49	0.00	0.37	1.00
*umc2298*	5	100–110	2	0.80	0.32	0.06	0.27	0.80
*umc1429*	5	235–260	2	0.60	0.48	0.00	0.36	1.00
*umc1153*	5	117–128	2	0.63	0.46	0.01	0.36	0.98
*umc1629*	5	120–125	2	0.70	0.42	0.00	0.33	1.00
*umc2143*	5	156–166	2	0.52	0.50	0.00	0.37	1.00
*umc1796*	6	127–141	2	0.64	0.46	0.00	0.35	1.00
*umc1831*	7	184–206	2	0.60	0.48	0.00	0.37	1.00
*umc1480*	7	100–930	3	0.66	0.49	0.02	0.43	0.96
*umc2392*	7	250–270	2	0.57	0.49	0.00	0.37	1.00
Minimum			2	0.41	0.28	0.00	0.24	−0.25
Maximum			3	0.83	0.65	0.82	0.58	1.00
Average			2.14	0.63	0.46	0.04	0.36	0.90

**Table 4:** Significant divergence (Fst) between groups (sub-populations) and average distances (expected heterozygosity) among maize populations and net nucleotide distance among sub-populations.

Sub-Population	F_st_ Value	Percent Mean Value of F_st_ within Population	Expected Heterozygosity	Number of Genotypes	Net Nucleotide Distance	
					Sub-Population I	Sub-Population II
I	0.2693	17.70	0.3729	17	0.000	-
II	0.1368	37.50	0.4497	36	0.098	0.000
III	0.3591	25.00	0.3111	24	0.125	0.113
Admixture	-	19.79	-	19	-	-

**Table 5:** Marker-trait association among the studied maize lines using mixed linear model (MLM) approach for the studied traits under Anand and Godhra environments.

Sl. No.	Markers	Traits	Chromosome	*p*-Value	R^2^
Environmental location: Anand					
1	*umc1429*	Days to 50% tasseling	5	0.01283	0.06847
2	*umc1429*	Days to 50% silking	5	0.0153	0.06507
3	*umc1303*	Number of kernels per row	4	0.00329	0.09578
4	*umc1303*	Number of kernels per ear	4	0.00289	0.09853
5	*umc2296* *	Protein content	5	0.00347	0.12996
6	*umc2050* *		3	0.00383	0.12748
7	*umc2038* *		4	0.00387	0.13098
8	*umc1303*		4	0.00431	0.08984
9	*umc1535* *	Total soluble sugars	2	0.00482	0.08726
10	*umc1303* *		4	0.00651	0.08107
11	*umc2296*	Carotene content	5	0.01202	0.1014
12	*umc1535*		2	0.01442	0.06541
13	*umc2296* *	Tryptophan content	5	0.00551	0.11606
14	*umc2136*		5	0.00732	0.10908
15	*umc2252* *		2	0.00888	0.10466
				Average	0.2546
Environmental location: Godhra					
1	*phi193225*	Days to 50% silking	3	0.0071	0.1136
2	*umc2384*	Ear height	4	0.0059	0.1435
3	*umc2380*	Ears per plants	2	0.0087	0.1583
4	*umc1941*		5	0.0100	0.1017
5	*umc2384*	Ear length	4	0.0155	0.1192
6	*umc1941*	Number of kernels per ear	5	0.0108	0.1000
7	*umc2038* *	Protein content	4	9.20 × 10^−4^	0.1728
8	*umc2050* *		3	0.0016	0.1509
9	*umc2296* *		5	0.0023	0.1383
10	*umc2380*		2	0.0114	0.1463
11	*umc1303*		4	0.0137	0.0662
12	*umc1535* *	Total soluble sugars	2	0.0058	0.0831
13	*umc1303*		4	0.0092	0.0739
14	*umc2296*	Lysine content	5	0.0155	0.0918
15	*umc2296* *	Tryptophan content	5	0.0032	0.1285
16	*umc2252* *		2	0.0096	0.1027
				Average	0.1182

* Common marker-trait association over both locations.

## Data Availability

Data will be made available upon request to the first author (R.P.).

## Supplementary Materials

The following supporting information can be downloaded at: https://www.mdpi.com/article/10.3390/plants13060823/s1. Figure S1. Agarose gel picture under UV trans-illuminant showing a model fingerprinting profile using SSRs (A) *umc2101* and (B) *umc2252*. Figure S2. QQ plots for MLM of the different traits evaluated at Anand environment. TA: days to 50% tasseling, SI: days to 50% silking, PH: plant height, EH: ear height, EPP: ears per plant, EL: ear length, EG: ear girth, KRP: kernel rows per ear, KPR: kernels per row, NKE: number of kernels per ear, Pro: protein content, TSS: total soluble sugar, Car: carotene content, Lys: lysine content, Trp: tryptophan content. Figure S3. QQ plots for MLM of the different traits evaluated at Godhra environment. TA: days to 50% tasseling, SI: days to 50% silking, PH: plant height, EH: ear height, EPP: ears per plant, EL: ear length, EG: ear girth, KRP: kernel rows per ear, KPR: kernels per row, NKE: number of kernels per ear, Pro: protein content, TSS: total soluble sugar, Car: carotene content, Lys: lysine content, Trp: tryptophan content. Table S1. Name and sources of studied maize inbreed lines. Table S2. Meteorological data for the experimental field (E1: Anand, E2: Godhra) across the cropping season from November 2020 to March 2021. Table S3. List of polymorphic SSR markers and their primer details for PCR amplification. Table S4. Mean values of the studied phenotypic and grain quality traits of 96 maize lines in two environments-Anand and Godhra. Table S5. Nei genetic distance matrix between studied maize lines. Table S6. The Evanno table output from STRUCTURE HARVESTER. Table S7. Q table derived from STRUCTURE analysis.
