# Supplementary material for: Genetic Diversity and Population Structure of Maize (Zea mays L.) Inbred Lines in Association with Phenotypic and Grain Qualitative Traits Using SSR Genotyping
Source: Plants (Basel). 2024 Mar 13;13(6):823. doi: 10.3390/plants13060823 (PMC10975177; doi:10.3390/plants13060823)
Supplement: Supplementary file 1 [file plants-13-00823-s001.zip › plants-2868639-supplementary.pdf]

Supplementary Figures

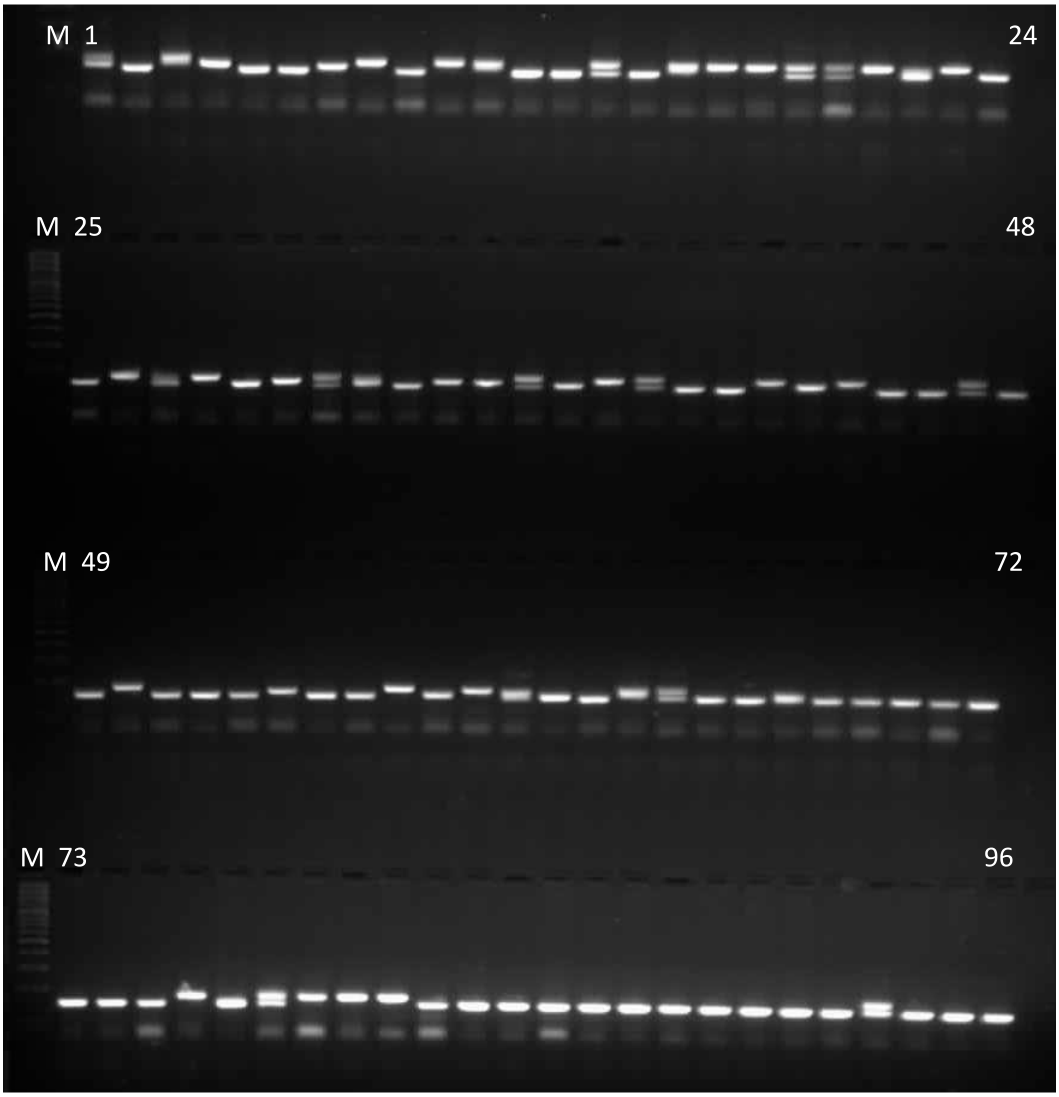

A

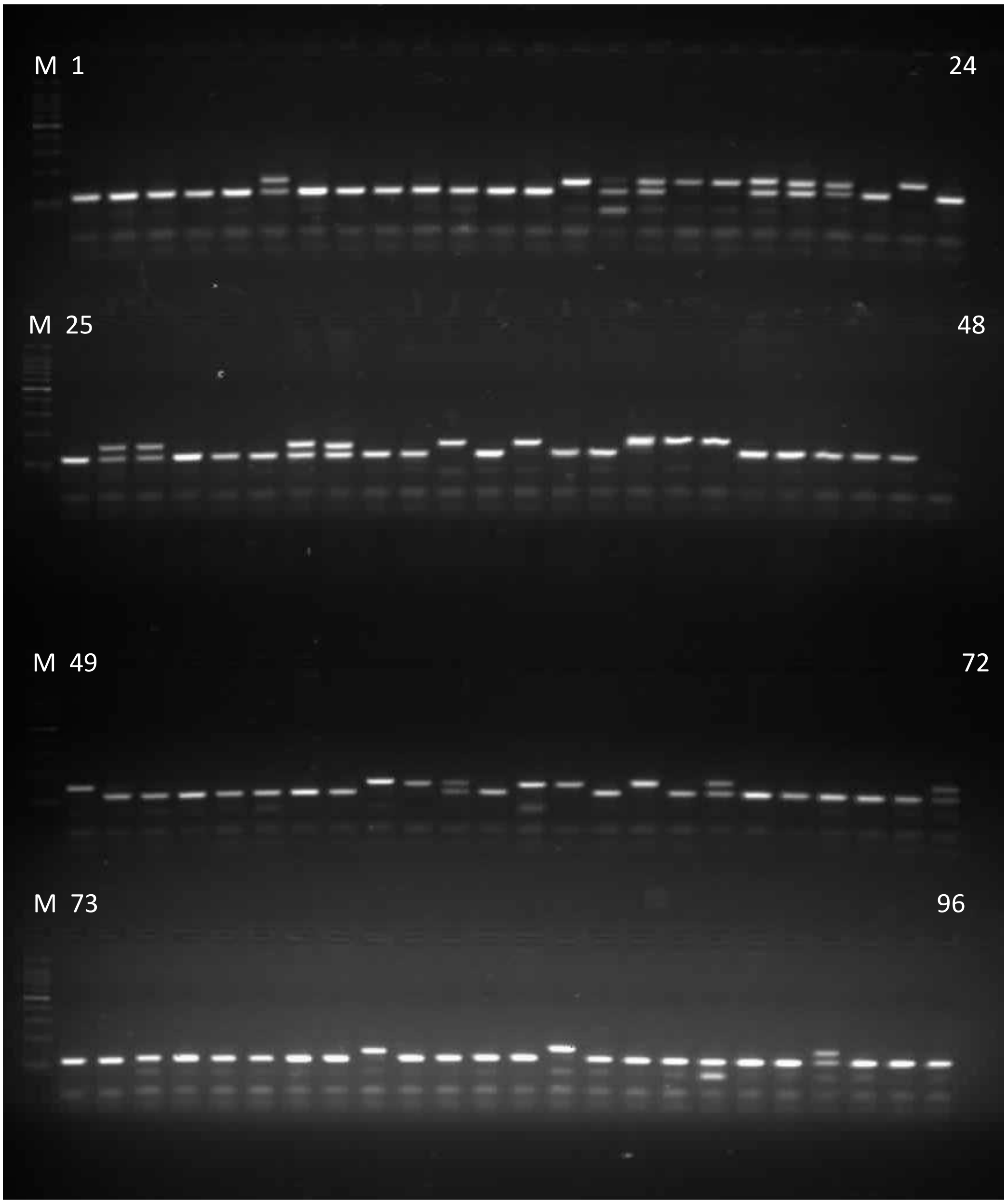

B

Figure S1. Agarose gel picture under UV trans-illuminant showing a model fingerprinting profile using SSRs - (**A**) *umc2101* and (**B**) *umc2252*.

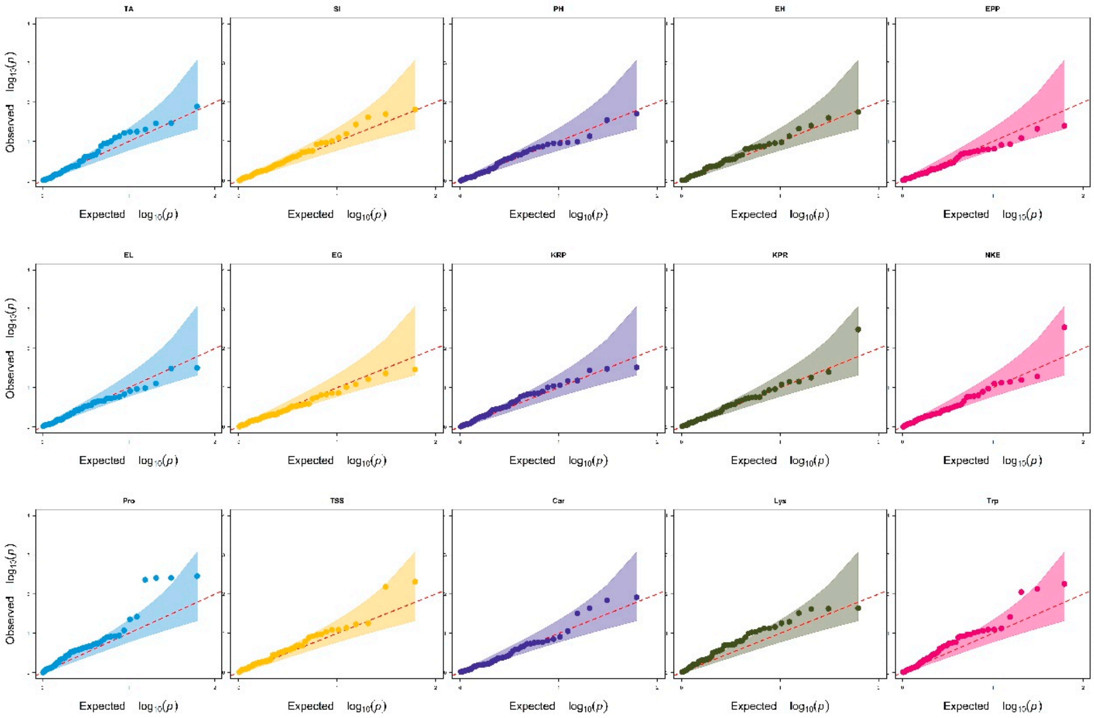

Figure S2. QQ plots for MLM of the different traits evaluated at Anand environment. TA: Days to 50% tasseling, SI: Days to 50% silking, PH: Plant height, EH: ear height, EPP: ears per plant, EL: ear length, EG: ear girth, KRP: kernel rows per ear, KPR: kernels per row, NKE: number of kernels per ear, Pro: protein content, TSS: total soluble sugar, Car: carotene content, Lys: lysine content, Trp: Tryptophan content.

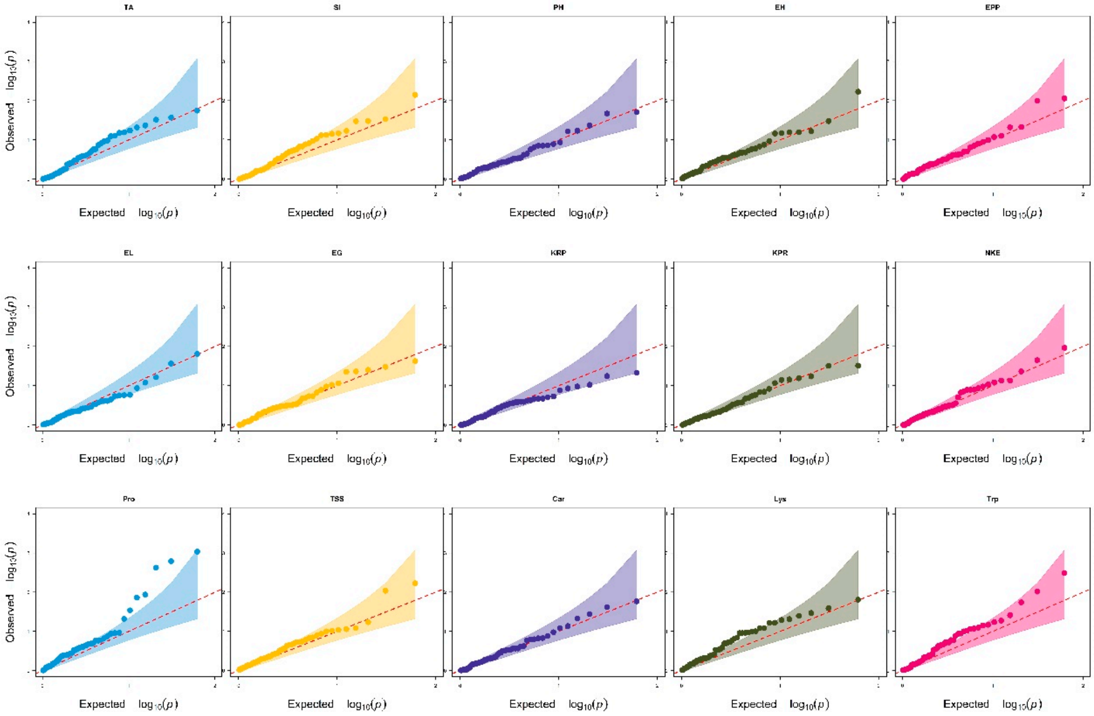

Figure S3. QQ plots for MLM of the different traits evaluated at Godhra environment. TA: Days to 50% tasseling, SI: Days to 50% silking, PH: Plant height, EH: ear height, EPP: ears per plant, EL: ear length, EG: ear girth, KRP: kernel rows per ear, KPR: kernels per row, NKE: number of kernels per ear, Pro: protein content, TSS: total soluble sugar, Car: carotene content, Lys: lysine content, Trp: Tryptophan content.

Supplementary Tables

Table S1. Name and sources of studied maize inbreed lines

Sr. No.	Genotypes	Source	Sr. No.	Genotypes	Source
**1**	I-07-14-1-2	AAU, Godhra	**49**	GWQPM-68-4	AAU, Godhra
**2**	I-07-28-3-2	AAU, Godhra	**50**	I-07-54-3-2	AAU, Godhra
**3**	I-07-29-1-3	AAU, Godhra	**51**	I-07-7-3-2	AAU, Godhra
**4**	I-07-66-1-2	AAU, Godhra	**52**	I-07-62-42-2	AAU, Godhra
**5**	I-07-66-2-3	AAU, Godhra	**53**	1820161/20-5	IIMR, Ludhiana
**6**	I-07-66-3-2	AAU, Godhra	**54**	1820164/20	IIMR, Ludhiana
**7**	I-07-66-4-1	AAU, Godhra	**55**	1820168/T1	IIMR, Ludhiana
**8**	I-07-56-4-3	AAU, Godhra	**56**	1820213/9-20	IIMR, Ludhiana
**9**	I-077-59-5	AAU, Godhra	**57**	1820215/C1-20	IIMR, Ludhiana
**10**	I-07-60-4-3	AAU, Godhra	**58**	1820228/T2	IIMR, Ludhiana
**11**	I-07-65-44-4	AAU, Godhra	**59**	11820211/T2	IIMR, Ludhiana
**12**	I-07-6-4-4	AAU, Godhra	**60**	1820211/T1	IIMR, Ludhiana
**13**	I-07-6-4-5	AAU, Godhra	**61**	1820200/C1-20	IIMR, Ludhiana
**14**	I-07-9-5	AAU, Godhra	**62**	1820198/C1-20	IIMR, Ludhiana
**15**	I-07-13-1-3	AAU, Godhra	**63**	1820197/T2	IIMR, Ludhiana
**16**	IL-14-28	AAU, Godhra	**64**	1820197/T1	IIMR, Ludhiana
**17**	IL-14-48	AAU, Godhra	**65**	1820167/C4-20	IIMR, Ludhiana
**18**	IL-14-60	AAU, Godhra	**66**	1820194/T1	IIMR, Ludhiana
**19**	IL-17-28	AAU, Godhra	**67**	1820192/4-20	IIMR, Ludhiana
**20**	IL-17-32	AAU, Godhra	**68**	1820229/T2	IIMR, Ludhiana
**21**	IL-17-34	AAU, Godhra	**69**	I-07-33-04	AAU, Godhra
**22**	IL-17-44	AAU, Godhra	**70**	I-07-34-3-1	AAU, Godhra
**23**	GYL-10	AAU, Godhra	**71**	I-07-36-1-4	AAU, Godhra
**24**	PFSR5-3-5	AAU, Godhra	**72**	I-07-36-2	AAU, Godhra
**25**	IL-15-39	AAU, Godhra	**73**	I-07-37-6-1	AAU, Godhra
**26**	IL-15-11	AAU, Godhra	**74**	I-07-40-4-2	AAU, Godhra

**27**	IGI-1101	AAU, Godhra	**75**	I-07-62-22-5	AAU, Godhra
**28**	IGI-1103	AAU, Godhra	**76**	1820162/T3	IIMR, Ludhiana
**29**	GYL-2	AAU, Godhra	**77**	1820166/T2	IIMR, Ludhiana
**30**	CML-307	AAU, Godhra	**78**	1820162/T2	IIMR, Ludhiana
**31**	IGI-1102	AAU, Godhra	**79**	1820162/T1	IIMR, Ludhiana
**32**	IGI-1104	AAU, Godhra	**80**	1820212/T2	IIMR, Ludhiana
**33**	LTP-1-1	AAU, Godhra	**81**	1820214/C1-20	IIMR, Ludhiana
**34**	LM-B-2	AAU, Godhra	**82**	1820228/T1	IIMR, Ludhiana
**35**	LM-5	AAU, Godhra	**83**	1820229/T1	IIMR, Ludhiana
**36**	H07R-1-3	AAU, Godhra	**84**	1820230/T1	IIMR, Ludhiana
**37**	GWQPM-5-3	AAU, Godhra	**85**	1820230/T2	IIMR, Ludhiana
**38**	GWQPM-11	AAU, Godhra	**86**	1820231/T1	IIMR, Ludhiana
**39**	GWQPM-17-2	AAU, Godhra	**87**	1820199/20-5	IIMR, Ludhiana
**40**	GWQPM-22-5	AAU, Godhra	**88**	1820194/T2	IIMR, Ludhiana
**41**	GWQPM-26-1	AAU, Godhra	**89**	1820196/T2	IIMR, Ludhiana
**42**	GWQPM-26-3	AAU, Godhra	**90**	1820193/T2	IIMR, Ludhiana
**43**	GWQPM-40-3	AAU, Godhra	**91**	1820195/20	IIMR, Ludhiana
**44**	GWQPM-40-5	AAU, Godhra	**92**	1820166/T1	IIMR, Ludhiana
**45**	GWQPM-46-2	AAU, Godhra	**93**	1820231/T3	IIMR, Ludhiana
**46**	GWQPM-47-1	AAU, Godhra	**94**	1820196/T1	IIMR, Ludhiana
**47**	GWQPM-55-2	AAU, Godhra	**95**	1820212/T1	IIMR, Ludhiana
**48**	GWQPM-67-2	AAU, Godhra	**96**	I-07-62-3-2	AAU, Godhra

Table S2. Meteorological data for the experimental field (E_1: Anand, E_2: Godhra) across the cropping season from November 2020 to March 2021.

Month-Year and Standard week		Sun shine	Rain fall (mm)	Wind speed (kmph)	Temperature (°C)			Relative humidity (%)		
**Month**	**Week**	**(hr/day)**			**Max.**	**Min.**	**Mean**	**Morning**	**Evening**	**Mean**
Environment 1 (E_1): Anand (22.54°N, 72.98°E, 45.10 m above mean sea level)										
**Nov-20**	44	9.1	0	2.4	34	17.6	25.8	80.4	30.5	55.5
	45	9.3	0	1.9	33.4	15.8	24.6	80.1	29.6	54.9
	46	8.5	0	2.6	32.6	19.1	25.9	78.9	39.6	59.2
	47	8.7	0	3.1	30.4	15.2	22.8	80.9	39.1	60
	48	8.4	0	4.3	30.4	17.6	24	76.6	41.6	59.1
**Dec-20**	49	9.5	0	1.5	32.7	15.1	23.9	87.9	36.1	62
	50	4.8	16.4	2.3	27.4	17.9	22.7	94.1	62.4	78.3
	51	8.8	0	3.7	26.6	12.6	19.6	82	44.3	63.1
	52	9.2	0	3.7	26.8	12.3	19.5	73.6	36.5	55.1
**Jan-21**	1	6.7	0	3.3	25.5	12.7	19.1	89.4	48.4	68.9
	2	8	0	4.6	26.6	13.8	20.2	79.7	44.1	61.9
	3	8.9	0	3.4	24.6	9.7	17.2	87	48.4	67.7
	4	9	0	3	27.8	13	20.4	84.4	43.7	64.1
	5	9.3	0	3.4	26.4	11	18.7	83.3	37.3	60.3
**Feb-21**	6	10.1	0	4.3	27.6	12.1	19.9	75	36	55.5
	7	9.4	0	2.8	32.3	15	23.6	79.3	32.4	55.9
	8	9.5	0	2.9	32	15.2	23.6	80.6	31.6	56.1
	9	9.3	0	2.4	33.2	16.5	24.9	82.9	30.6	56.8
**Mar-21**	10	9.5	0	4.2	31.2	16.5	23.9	80	31.3	55.6
	11	9.3	0	3.8	31	15.8	23.4	64.9	20.4	42.6
	12	9.5	0	3.1	35.6	20.3	28	77.1	31.9	54.5
	13	7.2	2	3.3	34.7	20.1	27.4	74.6	33.6	54.1
Environment 2 (E_2): Godhra (22.45°N, 40°E, 119.40 m above mean sea level)										
**Nov-20**	44	9.1	0	2.6	34.3	16.6	25.45	25.4	61.4	43.4
	45	8.9	0	2.2	33.6	14.5	24.05	24.1	63	43.55
	46	8.2	0	3.4	33.3	16.1	24.7	24.7	65.3	45
	47	8.8	0	3.1	30.6	13.3	21.95	22	71.1	46.55
	48	8.5	0	5.3	30.9	12.9	21.9	21.9	61.7	41.8
**Dec-20**	49	9.3	0	1.9	33.8	13.6	23.7	23.7	65.9	44.8
	50	4.9	10.5	2.4	27.6	12.9	20.25	20.3	83.7	52
	51	8.3	0	4.2	26.9	11.4	19.15	19.1	68.7	43.9
	52	9.1	0	4	26.7	10.4	18.55	18.5	76.4	47.45
**Jan-21**	1	6.8	0	5.3	24.1	12.1	18.1	77.9	58.4	68.15
	2	6.7	0	4.6	26.3	11.2	18.75	57.3	47.2	52.25
	3	6.2	0	3.5	24.1	7.9	16	76.8	47.9	62.35
	4	7.9	0	3.6	28.4	11.3	19.85	80.8	44.2	62.5
	5	7.2	0	3.9	26.3	10	18.15	67.8	45.1	56.45
**Feb-21**	6	9.2	0	4.4	27.9	9.3	18.6	73.8	49.5	61.65
	7	9.2	0	2.4	32.2	13.9	23.05	64.8	33.9	49.35
	8	9.3	0	3.2	32.2	14.6	23.4	63.3	35.6	49.45
	9	9	0	2.7	33.4	16.8	25.1	67.6	30.2	48.9
**Mar-21**	10	9	0	4.6	30.4	15.8	23.1	69.6	43.4	56.5
	11	9.4	0	4.6	30.6	13.6	22.1	47.8	29.3	38.55

12	8.2	0	2.9	36.2	17.9	27.05	62.7	28.1	45.4
13	8	10	3.4	35	17.4	26.2	66.9	33.4	50.15

Table S3. List of polymorphic SSR markers and their primer details for PCR amplification.

Sl. No.	SSR loci	Primer	Sequence 5'-3'	Repeat motif [1]	T_m
1	umc2244	F	TGTGAGCCTGATTCCATTTTGAC	$(GGC)_4$	67.4
		R	CATCGTGGGGACTAAAGCGAC		55.7
2	umc1756	F	ATCTCAGGTACTCTGCCTACGGG	$(CGG)_4$	67.3
		R	AACAGAGGGTAGCTTGTGGCCT		60.6
3	umc1552	F	CTCGATAGCTCTGCTGCTTCCTC	$(GGA)_7$	67.6
		R	CAACACCAGCCCTACCCAGA		59.3
4	umc1262	F	GTGAAGCTCTGCACCACGCT	$(GTC)_4$	68.9
		R	ATCGTCCAAAGAAGAAGAGGGAGA		57.3
5	umc2193	F	CCGAGGCATACGGACAATACC	$(TCC)_6$	69.3
		R	GTAGGAGGACGGGTGCTGGT		57.6
6	umc1922	F	CTTTTCCGCTGCTCTTCCTTTT	$(ATA)_6$	66.6
		R	TTCCCTTGTTCCAGATCCTCAATA		55.6
7	umc2125	F	CAAGGGTAAGGGCAAGATGGTAGT	$(GAG)_7$	67.5
		R	CTGAGGTCTACCTCGGCCATC		59.0
8	umc2252	F	CACTGCACTGCAAGGTACATACG	$(TCC)_4$	66.7
		R	GTCTTTGACCCCTTCCTCTTCTTG		58.1
9	umc1535	F	CAAGGCACCCACACACATACATA	$(AT)_7$	65.8
		R	GGCAGAGAGATGAAAAAGAATGGA		57.2
10	umc1831	F	TTTCGACTGCTAGTGTACTTGGGG	$(AG)_8$	65.6
		R	CTCTACATCTTCAGCGTCTCCACA		55.3
11	umc1285	F	TAAATATACGGCCCCAAGAAAACC	$(ACG)_4$	65.0
		R	AAACTGGATATGGTTGGTTGGTTG		55.8
12	umc2220	F	AATACAAGAAGCGAAAGGGGAAAG	$(ACT)_4$	66.1
		R	GCGTAGCAAACCAAAGAAGAAGAA		55.5
13	umc1497	F	GAGGAAGACGGAGACGGCAC	$(GAC)_4$	68.5
		R	TAGACGAGATCCTCTGAGGCAATG		57.8
14	umc2380	F	ATTTCCAGAACAGACCGGAGAAG	$(GCT)_5$	65.9
		R	ACATTGAGCCTAAGCGAGTCGT		58.5
15	umc2129	F	ACGTGGTCATCACTCACCGC	$(CGC)_5$	67.6
		R	AAGGAGGAGCGTTCTCGTGG		60.0
16	umc2077	F	AAACTCACTGAACATGATCCTGGC	$(AGC)_4$	66.1

		R	CTGGTTCGGATGCAAGTAGTCAG		57.5
17	umc1256	F	CATCTCGACCTTTGACATTCTCCT	$(CAT)_5$	66.4
		R	AGAAGACGATGATGATGATGCAGA		55.9
18	umc1230	F	GCGATTTCAACTATTTGTGGTAAAGG	$(TAA)_8$	65.1
		R	GTACGACCGTTGAAACTGTTGTTTT		56.3
19	umc1704	F	TTCACCGGGTAGTCCTTCTTACTG	$(AGG)_4$	66.2
		R	AAGTACGCTGTACGCAGGCAG		59.7
20	umc2214	F	ACCCCCTGATTCTCTCTTACGTTT	$(CTT)_4$	66.4
		R	CTGGATGAGGAGGAAGAATACGAG		56.3
21	umc1696	F	CTAGGGTTTAACCAACGGGGAG	$(GA)_8$	67.0
		R	TAAGGAGAGGGTCGATGAACACAT		57.4
22	umc2104	F	CTGCTGGCAGTGGCAGTATTC	$(CGT)_4$	67.8
		R	TACTGCTACACCTTTGTCGTCACC		58.6
23	umc1746	F	ACACGAGCATCCTACATCCTCCTA	$(CAC)_4$	66.9
		R	ACCTTGCCTGTCCTTCTTTCTCTT		58.3
24	umc1641	F	CTCCCTTCGTCTCCCGACTC	$(TCGCC)_4$	68.1
		R	CAGATCGGCTCAGCCACAAC		58.6
25	umc1010	F	TCCATGTATGTGTGTGTGACGTG	$(GA)_{10}$	66.0
		R	AAACCAAACAGCCAAAAGGACA		55.8
26	umc1361	F	GTGCTTCTTCCTGCCGTACTCC	$(TGT)_6$	68.1
		R	AAGAGCTACTTCAACAAGTCCGCC		59.1
27	umc1813	F	CTGTACATGGATATGGCATTGGTG	$(CAG)_8$	66.0
		R	GCATATACACCACCTTGGACAACA		57.2
28	umc1136	F	CTGCATACAGACATCCAACCAAAG	$(GCA)_5$	67.3
		R	CTCTCGTCTCATCACCTTTCCCT		56.1
29	umc1578	F	CGAGCAGCTAAGGTAGAGCAGCTA	$(GCG)_4$	67.9
		R	AAGCACTTCCAGTGGTACATGAGC		59.2
30	umc2277	F	TAACTGCAGAAACGGTGGTCAATA	$(ATA)_6$	66.7
		R	CTCTTCACGCTCAATAAACCCAGT		56.4
31	umc1273	F	GTTCGCTGCTGCTTCTTATATGCT	$(AAG)_4$	66.8
		R	AATTGGCGCAGGCTATAGACATTT		57.2
32	umc1521	F	GTCTCTCTCTTGCATGCCACTAGC	$(AGT)_4$	67.6
		R	GAGTCAGCTTCACCTCTCGATCTC		58.4
33	umc2174	F	GTACGTACGCAGCCACTTGTCAG	$(CGA)_4$	65.6
		R	ACATAAATAAAACGTGTGCCGCAG		59.9
34	umc2276	F	CTAGGTAGCCAGCTAGGTACGGGT	$(GGC)_4$	66.6
		R	AGTGGAGCTTCTTCATCCTACCG		61.2
35	umc1915	F	AGACGAGTTAAACCTCCATCATGC	$(ACA)_6$	65.9
		R	CCTACCCCAACTTGCTTGAGACTA		58.2
36	umc2272	F	GGAGGAAGCACAAGTACATGCAG	$(GCG)_5$	67.1
		R	CCTGAACTTGGTCTGCTTCTCTG		57.5
37	umc2050	F	CTCCTGCTGTGATTCTAGGACGA	$(CGC)_4$	67.2
		R	CTGGATCTCGGCATGGTCTT		56.9

38	umc1489	F	TTAATAGCTACCCGCAACCAAGAA	$(GCG)_5$	65.7
		R	CTGAGCCACAGTACCTTGCTGTT		59.3
39	umc1690	F	ACCTTAGTTACACAGGCACACGGT	$(GCA)_4$	67.6
		R	GGTGATGGGATTTTCGCATTATTA		53.6
40	umc1404	F	GTTGGCCTTCCTCTCTACCCC	$(CCG)_4$	68.2
		R	CCAGTTCATCAGGTCATCAACCTT		57.0
41	umc2258	F	GAATAAGACCAGACAGCACCGAAC	$(GTG)_5$	65.4
		R	AAGATTGTATAAATGGCAGCCACG		57.6
42	umc2259	F	GGCTCGACTTCGAGGACACC	$(CCG)_6$	68.5
		R	GAGGAGGAGAGGGACAGGGAAG		60.0
43	phi193225	F	GCTCTTGGCGTGCTTCTT	AAC	67.5
		R	GCGGGGAGGTGAAGAGCTA		59.1
44	umc1814	F	AGAAGAAGAGGAGGTTCCATGACC	$(CGA)_4$	66.4
		R	GCATGTTTCCCTTTCACCTCC		56.4
45	umc1458	F	CCAATAAACAAATCATCTCCCCCT	$(GCT)_5$	65.9
		R	TGCTATGCTATGTACAGGGACAGG		58.1
46	umc1057	F	GCCACGCTCAACTACGACAAC	$(CGG)_6$	67.7
		R	GAACCCCTCCACGTAGCTCAG		59.4
47	umc2101	F	CCCGGCTAGAGCTATAAAGCAAGT	$(AG)_7$	67.3
		R	CTAGCTAGTTTGGTGCGTGGTGAT		59.0
48	umc1780	F	CTGTCCCCAGGTTGCTGTAGTAGT	$(ACC)_4$	68.1
		R	CATGATGTACCCGCAACAAATG		54.8
49	umc2377	F	CCTTCAAACCAAATGTACAGCAGC	$(GAC)_4$	68.0
		R	CTCCTCAACGACAGCGTGTACC		56.9
50	umc2103	F	CCTATCCATGCTTGAGGTGTCG	$(GCG)_5$	67.1
		R	GTCCAGGAGGTTGTCGTCCA		59.1
51	umc1855	F	CTCCGGGCAGGTCCTACTACAT	$(GCA)_4$	68.5
		R	GATGAAGGAAAGAAGACACCATCG		55.6
52	umc1669	F	ACGAGGGCTTCTTCTCTGAGC	$(AGA)_4$	67.3
		R	GTTTCCTTCTTCATGCGACGAC		56.3
53	umc1757	F	TTTTCTGCAGGGATAACATTTGTG	$(TCC)_7$	64.5
		R	ATAGGAGGTGAGGTGAGGAGGAAG		59.2
54	umc1758	F	CTTCCTCCTCACCTCACCTCCTAT	$(CTT)_5$	67.7
		R	GGTAGCCAATCCTTCCTTCCTATG		56.9
55	umc1228	F	TCCTCAAGGACCTGCTCCAC	$(CAG)_8$	67.5
		R	ACCTATACAGACGGAGACGGGG		59.8
56	umc1294	F	GCCGTCAACGGGCTTAAACT	$(GAG)_4$	67.9
		R	GCCTCCAGCTCTCTCGTCTCTT		60.4
57	umc1390	F	CCTCGAAACAGATGCCTGAGTC	$(CCT)_4$	67.1
		R	AAATGATCCCGAAGCCTGAGAC		57.1
58	umc2061	F	GTCTGGAGAACTCCCTACCCATTC	$(CTG)_8$	67.2
		R	TAGCTTGAGAGACCGGAACAGC		58.7
59	umc2282	F	GAGACGGACTACTCGTACCCACAC	$(TGC)_4$	68.1

		R	CTCTGGAGCCTTCTCTTCCTCCT		60.2
60	umc2038	F	ACAGAAACCAATGCATGTGATGAG	$(GAC)_4$	65.5
		R	TGCATGGTTGCTTCAGCAGT		57.8
61	umc1620	F	CCACCGAGTGACTAGTTGTGAGAG	$(TTC)_4$	67.1
		R	CCTTTCAATGTTCATGTTCTCTTCC		54.3
62	umc1847	F	GCCCAAGGTAGATTTTTACTCTCCA	$(CGC)_6$	66.3
		R	AAGTCGTAGAGCTCGTCGTGATG		58.4
63	dupssr34	F	TCAGTGCTTTCATTGTAACGA	$(TTG)_{14}$	64.3
		R	ATAAACATCTTGCCAGCAAA		50.4
64	umc1775	F	GAGGACAACGCTGCTATTCTCG	$(CGC)_5$	67.1
		R	GGAACTCCGTCAAAATCCCATC		55.9
65	umc2404	F	ATGTATCATTGATTGGCAAACAGG	$(ATT)_4$	64.6
		R	ATCAGAGAGAAGGAAGGAAGAGGG		57.4
66	umc1313	F	GCTGTCTGTGACCAAGTTTCCTCT	$(CTT)_5$	67.5
		R	TGAAGAACAGGGACGTGATGATAA		55.8
67	umc2384	F	CTCTGGACTCGGTTCCTCCTTAC	$(GCC)_5$	67.3
		R	CTCCTCTTTCCTGATCCTCTGCT		57.9
68	umc2137	F	ACCACTGCAACCTAGAGCTGTACC	$(CGC)_6$	67.4
		R	CACTTGGGTTGTTCCACAGGAG		60.5
69	umc2200	F	CTTCCTCCTCCTCGTCTCTTTCAT	$(CGC)_4$	66.8
		R	GGGGCCAAATCTGAATCTTCC		57.7
70	umc1623	F	TCCAGCAGAACACCAACCTATTAGA	$(CGC)_4$	66.1
		R	GAGACCAGCAGGTAGTTCTTGGAA		58.4
71	umc2139	F	ATAAGGAACATCCCCACCTGTTTT	$(GCC)_4$	68.2
		R	GGTGTGCTGGGTTCTTGTGG		56.5
72	umc1238	F	AAACCAGTACACAGGAGGTTTTGC	$(TTC)_5$	65.5
		R	CTTTGGTGAAAGCTGCAATGTAGA		56.2
73	umc2360	F	TAGCAGCTAGCTTCAGTCACAGGC	$(GCC)_4$	67.2
		R	CAGATCGGACTACTGGTGGCTAAG		60.7
74	umc2400	F	TTTGGTGAAAGTGAAACCAAAGGT	$(AGA)_4$	65.4
		R	CTAGCTAGCTTCCTTCCTCCTCG		58.2
75	umc1761	F	GGCTTGTAGTTGGAGTGGTCGTAG	$(GCA)_5$	67.4
		R	AGCAGCTTCAGAGGAGGAAGAAG		58.5
76	umc2063	F	GGACTGAAGCGTGGAATGTTCT	$(AGG)_4$	67.2
		R	ATCGCAATCTGAGACCACTTGTT		56.7
77	umc2295	F	CTGCTTCCAACTTCCGTTGC	$(GGA)_5$	67.3
		R	CACCTTGAAGACGTAGTCCACCTT		57.0
78	umc2298	F	ATCCACTCCCAAGTCCCAACAC	$(GCG)_4$	67.0
		R	CTTCTTCCGGTTCTTCTTCAGGC		59.2
79	umc1429	F	GGGCCCTGTTAATCCTCATCTG	$(TGC)_5$	67.1
		R	TCCTCCTTTCTCTCATGTTTCTCG		56.2
80	umc2388	F	GTGAGGGACTGGAAGGAGGTGTA	$(GCC)_4$	67.3
		R	CTTCCATCGCTTTCGCGTACT		59.9

81	umc1597	F	ACAACCGCTGCGTAAACGAAT	$(CCT)_4$	66.9
		R	AGAGATTAGGCGAGCGAGGG		58.7
82	umc1564	F	AAGAAGAAAGAGAAGAAGCACGGG	$(CAG)_5$	65.9
		R	GGACAGCTCGTATTATAACCTGCG		57.3
83	umc2296	F	TGCAGTGACTTGAGACCATACACA	$(AGT)_4$	66.7
		R	GCTATACGCGTGCCAAGCTAAATA		58.1
84	umc1373	F	ATGATGATGACGACGACGAAGAT	$(AAG)_4$	65.6
		R	CGTCAGGTCTGTTGCATATCTGTT		57.1
85	umc1110	F	TTACACCAAGGTCCGAAACAAGAT	$(AGC)_7$	65.7
		R	TCTTGGAAGGCAAGACTCTACCTG		58.2
86	umc2373	F	ACCCAAGTGAGGTGAAGTGAAGC	$(GCT)_4$	67.3
		R	TATGGTACAGGCACAGCAGCAGTA		59.3
87	umc1482	F	GAACAAAGAATCACAACACGATGC	$(AGC)_5$	65.5
		R	CAGGTTCTGAGGAAAGCAAGGTT		57.4
88	umc2046	F	CGTTCACCTCTGCCTTTTGTTC	$(CCG)_4$	67.1
		R	GAAGGATCCGACGAAGACCTG		57.8
89	umc1171	F	ACGTACTACAGATAATGGGCGACG	$(GTT)_4$	66.3
		R	CGCCGTACCCATGAGTATAATGTAA		56.3
90	umc1629	F	GCTCCAAGTTCGTCGTCGTC	$(GAA)_5$	67.6
		R	CTCACGACCTTCTTCTTGCACAC		58.0
91	umc1060	F	ACAGGATTTGAGCTTCTGGACATT	$(CGG)_5$	65.9
		R	GGCCTCTCCTTCATCCTATTCAA		56.2
92	umc1752	F	ATCCTCCTCCATATTCTATCGCGT	$(CGG)_5$	67.4
		R	GAAACAGAGCAGGAACCGGAG		57.1
93	umc2201	F	AGGGAAGGGGAAAAGCAGTTAAG	$(GCG)_5$	66.2
		R	TAGAACGGCGAACAGAAGCAG		57.3
94	umc1941	F	ACGACGAGACTCTGTTCTGGTTCT	$(CTG)_{10}$	67.0
		R	AGGAGGATTACGTCAATCTGTTCG		56.4
95	phi087	F	GAGAGGAGGTGTTGTTTGACACAC	ACC	66.8
		R	ACAACCGGACAAGTCAGCAGATTG		59.6
96	umc2143	F	ACACACAACAGAGCCTTTTGTTCA	$(TTC)_4$	65.5
		R	AAGAAAAGGACACCAAACCAAACA		57.4
97	umc2136	F	CCAGATGCGGAAGTAGACGG	$(CCT)_8$	67.4
		R	GATTCGGAGGTGATCTGACCTGT		57.6
98	umc2307	F	GTCGACATCGTCTTCCCCAAG	$(CAG)_4$	68.2
		R	GTAGGAAGCCACGTACGGCTC		57.6
99	umc1153	F	CAGCATCTATAGCTTGCTTGCATT	$(TCA)_4$	66.1
		R	TGGGTTTTGTTTGTTTGTTTGTTG		53.7
100	umc1290	F	CTGCTCACGCTCATCCTCCT	$(GCG)_6$	68.4
		R	AGAGATTCATCAGAGTGGCGATG		56.5
101	umc2189	F	CGTAAGTACAGTACACCAATGGGC	$(CAG)_4$	66.7
		R	ACACCGACTACAAGCCTCTCAACT		59.8
102	umc1737	F	ATGCTTCTCTTCAGAAGCCATCC	$(AGA)_7$	66.2

		R	TAGCTAGGTAGTGATGTGCGTGCT		59.6
103	umc1534	F	GACCCCAAATCTCTCCTTCCTC	$(AAG)_5$	66.9
		R	TAGCTAAGCTTGTGCTTGCTCG		57.5
104	umc2100	F	AAAGGCATTATGCTCACGTTGATT	$(ATT)_4$	65.4
		R	TGACGTGCAAACAACCTTCATTAC		56.2
105	umc2029	F	AGAGCAATCCTGTTCCAAATGAAG	$(AGA)_5$	65.3
		R	CTTAAACCCAATCGAACTCGAACA		55.3
106	umc2240	F	CGCCTTTGTAACCCAGACTCATTA	$(AC)_6$	65.7
		R	CGGATGTTGCCAAGTACATCATATC		56.9
107	umc1838	F	CTTCACGAAGGTCTGAAACTTAGG	$(TA)_8$	65.6
		R	GTGCATAAATAAAAGGGGGAGAGT		55.0
108	umc1848	F	TCAGCTTTATCTCTACCCATTGCTTT	$(AG)_8$	65.3
		R	TCCATCATCTCCCTCCAGACTTTA		56.8
109	umc2223	F	TTTTGGGACTGAAGAAGAAGATCG	$(GCG)_4$	67.8
		R	ACTTCTGCAGAGCGAGCAGG		55.0
110	umc1122	F	CACAACTCCATCAGAGGACAGAGA	$(CGT)_7$	67.0
		R	CTGCTACGACATACGCAAGGC		58.3
111	umc1942	F	GAGCCCAAGATCATCACGGT		66.9
		R	TCGTCCTCATCTTCCTCCTCTTC		58.4
112	umc2151	F	ATATGTGGTATTTTCTGCAGGCGT	$(CAG)_4$	66.0
		R	AAAATCCTATACAGAAAACGGGCG		55.5
113	umc2083	F	GATGCTCAAGGAGCAGCGAC	$(CGG)_7$	67.9
		R	CAGGTGGTACGCCATGAACC		58.4
114	umc1446	F	GCGCTGCTGCTTCTTAAATTATCT	$(TAA)_7$	66.1
		R	GATGAGACCACCTACAAGTTCGCT		58.5
115	umc1558	F	GAGGTTGAGAGCAGCATATGAAAAA	$(AG)_7$	65.8
		R	AAGGTGGAGAACCAGAAGAGGAAG		58.2
116	umc1770	F	GAGGGATCATGGCTCTCTTCC	$(GGC)_4$	67.1
		R	GTCCATCATCAGCCTGTCACC		57.8
117	umc1849	F	TCCTTGTTGAAGATTTTATTTCTGCT	$(TA)_8$	63.9
		R	GGCTTTAAGTGATGCTCAAACGTA		55.6
118	umc1452	F	GATCCTAGCCTTGAAGGGGAACT	$(GCC)_4$	67.5
		R	AAGAGGAACCATTCTGCTATCGTG		56.7
119	umc1812	F	TACAAGGAAGGCAAGTTCATCCTC	$(ACC)_6$	65.7
		R	ATGCAGGTGACATTCATCATCATC		55.5
120	bnlg1803	F	GTATGCGTCGCTAGTCGTGA	$(AG)_{15}$	67.1
		R	TGTTGTCTATTGGCAACCGA		54.4
121	umc1353	F	AGACAGGATCATCGAAAACACACA	$(AAC)_4$	65.7
		R	ACCTCAGCCTCCTCGTCAACTACT		61.1
122	umc2191	F	CAGGTGGTGCAGAGTTTCACAT	$(CTC)_6$	67.4
		R	AAGGTGGAGGATGACTCCAAGAT		57.6
123	umc2204	F	AGCTGCTGAAGATGAAGGACAGG	$(GCG)_6$	67.2
		R	TCACCGTCGAGAACGACGAC		59.0

124	umc2226	F	TGCTGTGCAGTTCTTGCTTCTTAC	$(TGG)_6$	66.5
		R	AGCTTCACGCTCTTCTAGACCAAA		57.8
125	umc1104	F	CAACAATTCCAATCATGGCACTAA	(GAT)	57.6
		R	GTAACTCTGGTGAACTCAGAGGGC		64.4
126	umc1257	F	CAACGGAAGTGGCTGTAGAGTTTT	$(CAC)_4$	61.0
		R	ACAGAGCATGTCAGGTATTTGCAG		61.0
127	umc1273	F	GTTCGCTGCTGCTTCTTATATGCT	$(AAG)_4$	61.0
		R	AATTGGCGCAGGCTATAGACATTT		59.3
128	umc1303	F	CTTGGTAGCTTCGTATTCGACGAG	$(CCG)_4$	62.7
		R	ATCCTAGGAAAGCAGGGAGGG		61.8
129	umc1312	F	AAAGTTACTGCTGCCAAAGCTGTC	$(CAC)_4$	61.0
		R	AGATCGAGCGGTGGATATGGT		59.8
130	umc1360	F	GCTAGTTGAGTTCGACACCAGGTT	$(ACA)_4$	62.7
		R	TGACTGTGACTGTGACTATGACCG		62.7
131	umc1367	F	TGGACGATCTGCTTCTTCAGG	$(CGA)_6$	59.8
		R	GAAGGCTTCTTCCTCGAGTAGGTC		64.4
132	umc1480	F	AATGAAGGTGGATGTGCTGCTACT	$(GAA)_4$	61.0
		R	CTTCCCCATCTCCTCTTGAAGATT		61.0
133	umc1521	F	GTCTCTCTCTTGCATGCCACTAGC	$(AGT)_4$	64.4
		R	GAGTCAGCTTCACCTCTCGATCTC		64.4
134	umc1714	F	CAAGGGCTCTTGCTCTTGAACTAA	$(AGG)_8$	61.0
		R	CGACGACCTTAATTGTGTTCCTTT		59.3
135	umc1796	F	CGCTGAGGCTTAAGATGGTGTT	$(CCT)_5$	60.3
		R	AACGCCTTTACGAGCACGAAC		59.8
136	umc2392	F	CAGAGACCTCGACTTCGACCAC	$(GGC)_5$	64.2
		R	CTTCTGCTTCTGCTCGACCTTCT		66.1

[1] Based on the information in MaizeGDB site

Table S4. Mean values of the studied phenotypic and grain quality traits of 96 maize lines in two environments - Anand and Godhra.

Environment	Maize lines	TA	SI	PH	EH	EPP	EL	EG	KRP	KPR	NKE	Pro	TSS	Car	LYS	TRP
Anand	1	75.00	81	132.1	47.21	1.17	14.05	11.50	13.665	17.84	243.45	8.43	0.43	0.65	1.19	0.165
Anand	2	76.50	83	90.125	35.26	1.65	13.80	13.25	14.25	18.13	258.17	6.59	0.44	1.605	1.55	0.28
Anand	3	77.50	84	160.15	69.93	2.00	14.00	13.75	14.665	26.92	393.72	8.20	0.465	0.96	1.11	0.275
Anand	4	73.00	79.5	105.35	39.75	1.10	11.84	13.62	14.5	21.17	311.67	8.43	0.5	0.49	1.31	0.19
Anand	5	71.00	75.5	132	51.67	1.00	14.79	11.26	12.8	29.32	375.10	9.29	0.465	0.39	1.48	0.245
Anand	6	72.00	76.5	90.615	41.45	1.42	12.13	10.20	11.665	14.67	171.89	9.10	0.26	2.19	1.13	0.2
Anand	7	71.50	78	157.65	74.88	1.66	11.59	14.30	14.165	25.00	349.50	9.91	0.305	3.32	1.38	0.145
Anand	8	72.00	76	142.55	59.57	1.34	14.34	9.77	13.665	32.16	440.35	8.17	0.39	2.245	2.00	0.205
Anand	9	63.50	71.5	119.2	40.16	1.17	16.28	10.75	14	31.16	437.22	8.98	0.405	10.31	1.34	0.12
Anand	10	68.50	73.5	85.15	23.42	1.83	10.75	12.74	14.5	20.83	301.93	10.75	0.42	13.86	1.10	0.17
Anand	11	75.00	82	66.555	30.39	1.50	9.12	11.95	14.2	22.08	313.13	11.35	0.33	6.33	1.28	0.3
Anand	12	67.50	74	121.1	47.81	1.50	14.71	14.44	15	28.59	429.67	11.58	0.24	9.2	1.30	0.235
Anand	13	72.00	78.5	96.92	35.67	1.84	15.00	10.25	13	30.55	398.10	11.34	0.405	10.31	1.35	0.295
Anand	14	69.50	76	121.5	43.24	1.35	13.25	12.84	14.5	27.00	391.00	8.30	0.7	5.285	1.89	0.39
Anand	15	70.00	77.5	119.1	52.24	2.00	12.95	12.99	14.815	23.00	339.93	9.53	0.7	5.85	1.60	0.295
Anand	16	63.00	71	150.3	61.08	1.66	15.00	12.03	14.49	29.97	435.42	9.41	0.565	10.1	1.46	0.31
Anand	17	72.00	78.5	112.45	50.79	1.17	9.75	9.76	11.75	19.15	225.13	8.63	0.555	3.925	1.99	0.55
Anand	18	72.00	79	149.85	64.44	1.50	15.75	11.79	15	28.34	425.12	8.97	0.43	13.185	1.43	0.345
Anand	19	71.50	79.5	108.75	46.82	2.00	13.34	11.83	14	30.34	424.45	9.83	0.46	5.3	1.12	0.305
Anand	20	70.50	76.5	84.75	34.48	1.00	11.81	12.02	12.5	17.67	220.48	10.51	0.315	6.745	1.44	0.35
Anand	21	73.00	78.5	131.725	46.38	1.50	12.67	11.12	14.185	21.84	303.91	10.02	0.425	5.845	1.15	0.195
Anand	22	76.50	81	131.325	53.35	1.50	15.17	11.55	15.165	29.33	445.76	9.63	0.54	9.49	1.62	0.355
Anand	23	67.50	73.5	108.75	45.41	1.20	14.59	11.30	14.675	29.67	433.29	10.50	0.61	5.83	1.63	0.295
Anand	24	75.00	80.5	140.115	59.34	1.60	16.42	11.74	13.255	33.50	445.25	10.99	0.3	5.895	2.09	0.51
Anand	25	71.50	77.5	123.425	54.19	1.00	11.29	10.58	13.695	26.34	357.98	10.38	0.34	8.29	1.80	0.325

Anand	26	70.00	76.5	147.9	46.68	2.00	18.42	11.29	13.235	34.00	448.92	8.54	0.38	5.74	2.64	0.635
Anand	27	77.00	83	111.45	50.19	1.33	15.75	8.80	10.265	24.08	247.71	9.96	0.41	7.92	1.52	0.305
Anand	28	76.50	82	149.55	56.13	1.83	16.95	11.96	14.165	24.40	344.92	10.46	0.33	1.85	1.38	0.28
Anand	29	70.00	76	139.525	44.93	2.00	9.83	11.50	14.34	21.50	309.11	9.31	0.325	11.35	1.70	0.345
Anand	30	65.50	71	97.55	27.70	2.00	9.95	8.07	9.135	14.98	136.67	8.16	0.86	10.33	1.46	0.305
Anand	31	63.50	70	123.99	47.06	1.00	12.12	10.45	13.165	19.34	254.28	7.50	0.055	1.425	1.66	0.3
Anand	32	65.50	73.5	134.485	47.93	1.33	13.06	12.08	14	28.00	392.00	8.60	0.43	0.575	1.61	0.385
Anand	33	71.50	76.5	122.4	44.77	1.00	12.16	12.64	15.775	29.80	471.24	8.88	0.42	4.33	1.65	0.395
Anand	34	74.00	81	126.4	38.47	1.50	8.95	11.75	16	17.50	270.56	8.74	0.48	14.805	1.83	0.395
Anand	35	72.50	78.5	129.85	50.80	1.66	13.20	8.65	11.65	32.10	374.28	10.92	0.63	9.26	2.53	0.63
Anand	36	74.00	81	117.9	57.40	1.50	12.42	9.57	13.17	22.00	290.05	8.65	0.515	9.26	1.65	0.38
Anand	37	61.50	67.5	127.7	38.55	1.33	12.00	10.59	13.67	22.49	304.55	8.38	0.33	2.64	1.85	0.405
Anand	38	66.00	72	117.325	41.60	1.00	17.06	12.47	14.995	30.83	463.40	10.93	0.55	0.245	2.01	0.48
Anand	39	64.00	71	96.45	43.79	1.50	12.00	10.98	13.94	13.67	193.65	11.07	0.52	2.66	2.21	0.545
Anand	40	65.50	71	154.75	67.94	1.17	16.50	10.01	13.155	34.75	458.67	12.86	0.27	0.115	2.12	0.68
Anand	41	62.00	68	130.975	34.71	1.83	14.13	8.67	9.76	26.75	264.60	11.76	0.8	0.48	1.94	0.5
Anand	42	68.50	75	146.05	58.43	1.17	10.20	9.57	13.065	23.84	311.73	13.58	0.52	0.13	1.87	0.635
Anand	43	60.50	67	128.65	51.75	1.00	16.08	10.62	12.94	24.28	314.08	10.72	0.5	0.21	3.16	0.755
Anand	44	55.50	62	125.3	52.48	1.50	13.95	10.48	12.875	23.50	302.83	14.76	0.55	1.17	1.67	0.545
Anand	45	56.50	63.5	141	52.76	1.00	12.34	9.45	11.995	19.92	238.70	13.10	0.61	0.145	2.00	0.5
Anand	46	59.00	67	117.475	51.36	1.33	15.27	8.81	10.405	22.66	237.43	13.44	0.3	1.34	1.90	0.485
Anand	47	62.50	68	143.9	56.01	1.33	15.00	9.00	12.66	29.84	376.29	13.83	0.31	0.275	2.17	0.47
Anand	48	72.00	76	149.55	72.06	1.50	15.99	14.08	13.535	24.84	335.58	14.22	0.635	0.22	2.03	0.51
Anand	49	67.00	73.5	172.05	78.97	1.67	15.00	13.84	12.47	29.50	369.27	12.45	0.475	0.23	2.92	0.73
Anand	50	65.00	72	144.7	64.40	1.83	13.50	8.63	11.675	29.33	341.61	13.81	0.455	13.84	1.36	0.305
Anand	51	64.50	70	104.90	40.77	1.33	12.09	9.47	10.63	25.16	269.38	15.34	0.46	4.29	1.72	0.45
Anand	52	71.00	78.50	113.80	49.64	1.17	12.08	12.21	14.17	29.83	421.54	15.31	2.32	3.34	2.12	0.535
Anand	53	64.50	71.00	112.75	32.79	1.50	14.52	9.88	13.00	24.50	318.00	16.77	1.62	2.19	1.92	0.705

Anand	54	69.00	75.00	119.80	51.72	1.50	15.68	13.42	16.35	36.65	600.44	16.31	1.73	6.35	1.91	0.47
Anand	55	70.50	78.00	137.95	77.03	1.00	17.65	14.50	12.10	33.99	412.01	15.38	1.88	1.80	2.64	0.61
Anand	56	72.00	78.50	151.68	67.69	1.00	16.92	13.54	13.67	37.18	507.52	12.01	1.00	3.94	3.02	0.63
Anand	57	71.00	76.50	90.44	22.69	1.68	12.25	13.17	15.34	30.16	463.11	12.45	2.44	8.43	4.27	0.985
Anand	58	60.50	68.00	95.22	59.10	1.83	14.92	12.83	13.00	30.83	400.34	13.22	2.26	4.99	2.58	0.605
Anand	59	76.50	82.50	83.60	40.64	1.50	14.78	12.50	11.96	22.60	270.54	15.74	1.74	3.15	1.56	0.49
Anand	60	73.50	80.00	134.20	39.31	1.50	15.67	12.17	17.18	32.00	550.58	13.87	2.28	6.83	1.35	0.355
Anand	61	70.00	76.50	103.88	27.63	1.00	16.49	12.09	13.84	33.89	470.04	12.94	1.70	5.34	3.23	0.72
Anand	62	63.00	69.50	138.23	63.49	1.42	17.08	14.25	15.17	36.65	556.25	14.37	2.16	18.81	1.52	0.33
Anand	63	74.50	78.50	92.25	21.58	1.33	14.00	12.25	14.50	24.50	357.00	14.13	1.66	18.53	1.73	0.33
Anand	64	72.50	79.50	126.87	41.87	1.20	17.33	14.17	13.67	34.00	464.34	13.93	1.34	33.64	2.75	0.46
Anand	65	65.00	70.50	126.25	46.67	1.67	9.97	13.88	15.65	25.15	393.90	13.90	3.24	13.80	1.99	0.54
Anand	66	64.00	70.00	137.50	55.07	2.00	9.88	10.71	15.27	21.75	332.20	13.11	3.13	6.81	2.41	0.48
Anand	67	71.50	79.00	79.95	31.45	2.00	14.67	12.75	15.50	29.23	453.38	15.21	2.44	6.33	1.49	0.31
Anand	68	76.50	81.00	89.09	41.40	1.50	11.88	11.00	12.17	17.32	210.86	14.87	1.58	3.82	2.65	0.615
Anand	69	73.50	80.00	108.17	32.22	1.33	12.92	12.25	13.67	27.28	370.11	11.59	2.22	24.85	1.87	0.41
Anand	70	71.00	76.50	114.74	29.69	2.34	12.92	11.00	13.67	27.84	380.67	12.33	1.65	17.34	1.42	0.325
Anand	71	73.00	78.50	96.80	36.50	1.17	11.17	9.17	12.34	18.50	227.78	12.23	2.06	3.01	3.16	0.725
Anand	72	71.50	77.50	102.68	48.00	1.33	17.00	15.02	16.00	32.00	512.00	12.87	2.25	3.94	2.16	0.605
Anand	73	64.00	71.50	108.98	36.65	1.80	18.27	13.33	11.95	21.50	256.53	12.31	2.23	3.34	2.18	0.515
Anand	74	69.50	77.00	121.79	45.19	1.00	13.38	13.42	12.67	26.67	337.78	13.70	3.20	5.50	1.82	0.465
Anand	75	64.50	71.50	129.57	47.47	1.50	15.66	12.71	16.50	34.75	573.75	15.13	2.21	4.33	1.06	0.21
Anand	76	69.50	74.50	133.89	61.99	1.67	13.98	13.98	15.50	18.84	291.84	13.10	2.45	3.11	1.22	0.31
Anand	77	71.50	78.00	108.77	54.75	1.00	13.70	12.75	15.67	30.00	470.11	15.41	2.08	4.85	1.62	0.595
Anand	78	74.50	80.50	128.84	47.44	1.00	12.17	13.34	15.50	26.42	409.92	13.67	2.06	6.33	2.02	0.46
Anand	79	75.50	81.50	136.75	68.86	1.00	12.96	13.84	15.34	28.00	428.67	15.66	1.54	6.35	2.85	0.36
Anand	80	77.50	81.50	173.43	69.48	1.00	15.82	11.75	13.65	36.06	492.90	14.73	2.31	8.28	1.67	0.415
Anand	81	74.00	79.50	116.06	40.57	2.00	13.75	12.00	14.67	28.50	419.00	15.43	2.55	4.42	2.45	0.7

Anand	82	74.00	82.00	88.81	29.76	1.50	10.25	9.67	13.00	19.92	256.34	15.01	3.27	5.86	3.16	0.745
Anand	83	77.00	83.00	107.94	57.56	1.00	11.63	8.75	12.84	28.17	361.45	16.12	2.02	7.33	2.52	0.75
Anand	84	73.00	78.00	101.91	35.27	1.00	11.34	11.33	14.00	22.33	312.67	14.80	2.07	3.44	1.72	0.5
Anand	85	75.50	82.00	103.87	45.67	1.50	13.58	12.50	15.00	36.82	551.41	14.19	1.51	7.93	2.14	0.73
Anand	86	78.50	85.00	108.41	45.69	1.00	12.08	11.00	13.00	22.00	286.56	14.27	1.13	9.74	1.23	0.325
Anand	87	70.50	76.50	114.03	44.22	1.50	15.82	10.75	11.84	31.14	369.69	14.14	2.13	6.34	1.79	0.43
Anand	88	78.50	85.50	91.93	21.08	1.00	16.87	11.25	13.50	32.14	434.07	13.80	2.57	14.79	2.21	0.59
Anand	89	75.50	82.50	115.80	42.68	1.33	8.80	11.75	17.50	14.30	250.60	15.32	2.26	4.81	1.74	0.4
Anand	90	73.00	79.50	105.94	23.11	2.00	12.17	11.08	12.82	25.67	329.14	14.43	2.15	14.95	2.65	0.68
Anand	91	74.50	81.50	95.09	24.45	1.50	11.83	11.58	14.34	25.17	360.42	15.55	2.21	17.10	2.75	0.565
Anand	92	73.50	78.50	141.15	36.57	1.33	12.44	11.25	12.84	26.59	340.81	13.62	2.03	5.98	2.40	0.6
Anand	93	72.00	79.50	92.78	30.09	1.17	12.89	12.82	13.50	27.01	364.94	12.55	1.42	13.80	1.74	0.405
Anand	94	72.50	80.00	110.55	42.06	1.00	14.01	12.78	14.50	28.00	405.50	14.10	2.57	9.86	2.57	0.64
Anand	95	71.50	78.00	131.33	34.13	1.50	14.67	11.84	13.67	30.16	412.60	14.49	2.61	7.48	2.03	0.54
Anand	96	74	78	131.775	58.57	1.33	13	14.625	15.665	29.5	461.335	14.89	2.355	7.89	2.22	0.715
Godhra	1	76.50	81.50	246.00	139.10	2.17	19.06	14.08	16.53	30.62	506.50	8.69	0.45	0.575	1.19	0.165
Godhra	2	73.50	79.00	137.65	77.92	1.17	14.81	12.03	16.74	21.30	355.86	6.72	0.45	1.295	1.58	0.29
Godhra	3	76.00	83.00	194.95	96.55	1.00	18.97	14.22	12.69	38.14	482.78	8.34	0.48	1.035	1.11	0.28
Godhra	4	74.00	81.00	198.65	107.80	1.50	13.90	14.50	17.52	29.17	511.62	8.46	0.52	0.24	1.32	0.18
Godhra	5	69.00	73.50	203.25	98.61	2.17	19.38	12.83	12.77	29.92	381.63	9.24	0.46	0.39	1.49	0.27
Godhra	6	68.00	73.00	163.03	100.04	1.17	12.63	13.35	14.72	22.78	334.98	9.82	0.28	0.93	1.13	0.21
Godhra	7	70.00	75.50	248.82	130.60	1.50	16.34	12.70	15.17	33.64	511.29	10.07	0.34	2.635	1.38	0.165
Godhra	8	69.50	76.00	138.33	62.32	1.83	11.08	9.95	12.53	20.20	253.63	8.78	0.41	1.72	2.00	0.22
Godhra	9	61.00	67.50	201.80	98.45	2.00	10.16	11.03	12.31	18.71	230.46	8.30	0.43	8.13	1.34	0.15
Godhra	10	67.00	72.50	127.13	48.32	1.00	9.84	9.74	16.99	24.18	410.01	11.06	0.43	13.155	1.10	0.175
Godhra	11	71.50	78.50	152.60	79.98	1.17	13.78	10.40	12.04	26.73	322.21	11.99	0.35	3.78	1.29	0.31
Godhra	12	68.00	74.00	181.00	101.03	1.33	13.51	9.49	13.96	29.39	409.86	11.10	0.25	8.415	1.31	0.3
Godhra	13	69.00	75.00	217.13	121.45	1.50	17.84	11.11	9.42	35.03	330.77	11.37	0.43	8.885	1.35	0.305

Godhra	14	68.00	73.00	177.95	67.79	1.50	13.21	11.65	13.83	27.46	378.93	8.16	0.72	8.265	1.88	0.4
Godhra	15	69.00	76.00	154.00	78.65	1.50	15.08	10.44	13.44	25.95	349.11	10.29	0.71	6.245	1.59	0.31
Godhra	16	64.50	71.00	152.30	74.03	1.83	15.31	12.12	12.95	21.46	279.18	10.92	0.60	8.89	1.46	0.315
Godhra	17	73.50	80.00	157.30	88.62	1.50	17.30	12.28	15.48	26.06	403.70	9.03	0.57	1.405	1.99	0.57
Godhra	18	68.50	74.50	223.38	112.33	2.17	15.95	13.75	14.41	28.43	409.69	11.50	0.44	10.04	1.42	0.375
Godhra	19	70.50	77.00	149.55	83.93	1.17	17.50	14.60	13.63	30.88	420.41	11.03	0.48	4.9	1.15	0.31
Godhra	20	68.50	76.00	262.70	137.48	1.50	21.67	14.40	13.91	28.44	395.86	10.61	0.34	4.92	1.46	0.355
Godhra	21	71.50	78.00	133.80	75.56	1.67	14.50	13.10	16.76	33.15	556.38	10.96	0.65	6.165	1.14	0.21
Godhra	22	73.50	81.50	202.35	121.53	1.83	14.96	12.43	13.54	21.58	292.28	11.19	0.55	7.315	1.64	0.38
Godhra	23	69.00	76.00	203.32	116.67	1.67	17.13	12.95	16.14	38.45	620.87	11.05	0.61	3.505	1.65	0.335
Godhra	24	73.00	78.50	200.45	113.95	2.83	18.54	11.89	12.86	34.20	439.17	12.12	0.30	6.755	2.00	0.525
Godhra	25	70.50	78.00	200.13	127.42	1.84	17.27	12.45	15.44	31.09	479.80	12.00	0.35	8.1	1.79	0.32
Godhra	26	70.00	75.00	168.03	80.24	1.34	14.67	11.40	13.59	25.04	340.61	12.03	0.38	4.055	2.67	0.655
Godhra	27	75.00	82.00	192.53	111.70	1.17	14.19	12.35	13.38	17.55	234.87	10.61	0.43	4.59	1.56	0.32
Godhra	28	74.50	82.00	120.03	54.13	1.17	13.63	12.40	14.53	27.73	403.52	12.09	0.34	1.55	1.39	0.29
Godhra	29	66.00	74.00	178.68	86.49	1.00	16.00	13.34	14.74	33.57	493.11	7.76	0.34	10.52	1.71	0.355
Godhra	30	66.50	72.00	146.89	81.02	2.00	10.50	11.50	14.64	23.10	338.77	7.24	0.87	6.59	1.47	0.33
Godhra	31	62.50	70.00	105.12	50.05	2.17	13.83	13.28	14.34	29.74	426.54	8.42	0.07	0.795	1.70	0.36
Godhra	32	65.50	71.50	183.75	94.12	1.50	15.72	11.95	13.59	32.93	446.81	9.36	0.44	0.56	1.62	0.41
Godhra	33	70.50	77.00	87.63	31.97	1.17	11.13	10.78	11.50	19.90	230.09	11.13	0.43	4.795	1.66	0.435
Godhra	34	72.50	79.00	154.65	73.49	1.83	11.22	12.70	14.22	22.44	319.31	11.10	0.48	9.1	1.86	0.39
Godhra	35	73.00	79.50	160.48	71.15	1.50	11.50	8.55	8.34	11.50	96.08	10.54	0.64	6.34	2.59	0.645
Godhra	36	71.50	78.50	186.80	82.16	2.00	16.33	12.29	12.39	29.83	368.87	9.67	0.54	7.875	1.67	0.38
Godhra	37	62.50	67.50	207.92	85.88	1.50	16.11	13.54	14.50	24.52	356.06	10.05	0.38	1.34	1.86	0.425
Godhra	38	62.00	69.50	114.69	70.01	1.84	11.20	11.98	14.89	25.13	375.58	11.17	0.57	0.415	2.02	0.48
Godhra	39	61.00	66.00	185.82	93.52	1.83	14.50	14.54	17.22	26.10	448.95	12.90	0.54	1.165	2.24	0.545
Godhra	40	64.50	71.00	230.92	105.48	2.00	17.38	15.75	13.13	29.05	381.69	14.25	0.28	0.42	2.14	0.69
Godhra	41	60.00	65.50	172.13	86.85	2.00	18.74	15.19	17.14	39.90	684.16	13.01	0.82	0.745	1.99	0.51

Godhra	42	63.50	71.00	115.60	49.13	1.33	15.00	11.58	17.64	30.54	541.98	15.01	0.55	0.32	1.92	0.64
Godhra	43	60.00	68.00	186.80	91.20	2.17	17.20	12.24	12.80	34.74	445.73	12.08	0.53	0.205	3.16	0.735
Godhra	44	57.50	64.50	203.82	96.97	1.67	15.89	15.08	14.53	25.57	371.94	14.02	0.57	1.325	1.67	0.565
Godhra	45	57.00	64.50	209.53	95.03	1.50	20.50	16.06	14.15	35.62	504.19	11.89	0.61	0.98	2.07	0.505
Godhra	46	63.50	71.00	151.60	82.04	2.17	15.92	11.50	12.18	18.75	229.29	14.55	0.34	1.285	1.95	0.495
Godhra	47	58.50	66.50	183.63	99.80	1.33	17.98	15.31	13.17	32.19	424.21	10.77	0.34	0.96	2.15	0.48
Godhra	48	71.50	78.50	120.82	60.30	1.50	13.00	9.68	14.97	21.06	316.11	15.78	0.63	0.49	2.06	0.52
Godhra	49	69.50	76.00	131.47	67.80	1.00	14.75	14.18	16.56	25.43	421.23	13.39	0.49	0.53	3.01	0.735
Godhra	50	63.00	70.50	187.48	74.47	1.84	11.09	14.31	22.18	22.11	489.51	15.98	0.48	10.355	1.36	0.32
Godhra	51	62.50	68.00	119.98	66.82	1.17	11.50	8.25	11.79	17.78	209.31	15.07	0.46	6.25	1.74	0.46
Godhra	52	67.50	75.00	154.49	65.49	1.33	14.00	11.40	12.56	24.25	289.83	15.30	2.36	2.89	2.16	0.55
Godhra	53	59.50	65.50	197.47	82.09	1.50	19.00	12.57	13.75	33.63	462.46	16.72	1.64	2.79	2.73	0.7
Godhra	54	65.00	72.50	142.99	77.95	1.00	15.40	12.42	16.13	29.21	471.80	17.22	1.78	6.78	1.93	0.475
Godhra	55	66.00	72.00	180.99	70.95	1.29	17.09	14.25	12.90	30.09	387.54	15.63	1.97	3.73	2.68	0.615
Godhra	56	70.00	76.00	99.14	49.31	1.33	12.45	9.35	11.57	22.13	255.00	12.08	1.10	5.11	3.05	0.65
Godhra	57	67.00	74.50	207.78	83.28	1.67	16.20	10.41	13.46	30.83	415.46	12.85	2.55	9.00	4.47	0.98
Godhra	58	61.00	68.50	185.50	65.94	1.67	18.20	13.40	14.57	35.87	513.18	13.35	1.96	4.99	2.80	0.52
Godhra	59	76.50	83.00	147.00	86.33	1.00	13.48	10.60	12.47	22.28	278.45	16.47	1.66	3.15	1.72	0.46
Godhra	60	73.00	80.50	209.17	103.45	1.15	17.90	12.90	12.14	32.54	388.18	13.83	1.21	7.16	1.32	0.31
Godhra	61	68.50	74.00	172.97	112.92	1.49	18.09	12.24	12.72	32.31	408.74	12.56	1.70	6.81	3.29	0.77
Godhra	62	64.50	71.00	190.00	87.83	1.33	19.62	13.13	13.51	36.61	491.02	14.14	2.11	15.59	1.58	0.33
Godhra	63	71.00	76.50	131.40	61.93	1.50	16.10	9.97	12.62	32.97	409.04	14.22	1.63	19.06	1.49	0.32
Godhra	64	71.00	78.00	197.95	105.39	1.17	19.08	13.65	14.04	41.33	577.50	13.77	1.18	35.72	2.28	0.455
Godhra	65	62.50	69.00	188.64	110.66	1.00	10.00	12.54	12.79	17.46	223.78	14.00	3.54	13.84	2.00	0.55
Godhra	66	61.50	68.50	89.83	50.83	1.00	7.45	10.02	10.51	23.03	242.88	13.41	3.34	8.87	2.29	0.475
Godhra	67	69.50	76.00	201.02	106.16	1.17	16.45	13.24	13.94	39.02	544.60	14.70	2.53	7.77	1.44	0.33
Godhra	68	74.50	80.50	156.68	65.60	1.12	16.19	13.25	15.41	29.00	441.08	14.93	1.62	2.46	2.91	0.63
Godhra	69	70.00	77.00	226.50	116.07	1.50	18.12	14.23	14.44	36.03	519.98	11.99	2.25	27.11	1.96	0.405

Godhra	70	70.00	76.00	140.32	73.66	2.00	12.08	10.94	12.76	28.33	345.23	12.05	1.91	16.82	1.64	0.365
Godhra	71	68.50	75.00	101.99	43.47	1.50	11.30	9.60	11.87	23.66	213.97	12.52	2.20	1.94	3.22	0.765
Godhra	72	69.00	76.00	198.59	104.99	1.39	19.18	12.65	13.95	34.96	486.69	13.27	2.05	2.81	2.17	0.49
Godhra	73	61.00	67.00	162.30	66.66	1.50	11.73	13.87	22.55	21.08	476.31	12.63	2.36	3.32	2.15	0.53
Godhra	74	67.00	74.00	161.25	65.49	1.33	14.94	13.47	13.83	27.61	381.02	14.17	3.18	6.33	1.89	0.495
Godhra	75	62.50	69.00	194.47	108.65	1.33	15.23	15.10	17.24	30.95	534.20	15.08	2.25	8.19	1.07	0.21
Godhra	76	70.00	78.00	150.45	86.33	1.17	12.80	12.70	15.17	26.45	377.93	12.68	2.43	2.19	1.26	0.31
Godhra	77	71.00	76.50	182.51	113.68	1.67	16.63	13.00	14.00	33.38	467.28	15.30	2.30	7.00	1.65	0.4
Godhra	78	70.00	77.00	161.49	69.65	1.63	15.64	13.94	12.06	22.09	266.24	14.00	2.28	4.15	2.03	0.455
Godhra	79	71.00	78.00	151.74	77.66	1.50	15.07	11.77	16.06	35.59	590.93	15.44	1.56	4.52	2.84	0.69
Godhra	80	75.00	80.50	174.47	85.49	1.82	11.53	10.73	12.32	18.83	224.19	14.73	2.33	9.56	1.61	0.405
Godhra	81	70.00	77.00	181.25	95.62	1.67	13.13	12.54	14.54	21.99	319.65	14.74	2.52	4.95	2.41	0.705
Godhra	82	69.50	77.00	159.62	87.85	1.17	14.55	13.14	16.82	29.72	480.38	15.08	3.23	5.33	3.03	0.765
Godhra	83	72.50	79.00	97.68	39.30	1.50	13.90	13.00	24.92	22.65	587.62	15.36	1.92	9.71	2.41	0.75
Godhra	84	72.50	79.00	129.12	54.33	1.00	13.32	13.80	15.26	26.17	399.65	15.13	2.01	2.35	1.76	0.495
Godhra	85	72.00	76.50	179.80	89.01	1.15	22.24	14.95	15.98	42.17	672.92	14.20	1.46	9.70	2.14	0.735
Godhra	86	75.50	81.50	123.10	30.56	1.58	9.30	7.55	11.19	19.47	216.53	13.59	1.04	8.87	1.31	0.315
Godhra	87	64.00	71.50	153.33	69.08	1.78	15.98	11.49	11.81	29.00	342.72	12.96	2.34	3.83	1.74	0.43
Godhra	88	74.50	82.00	202.49	98.65	1.17	13.78	11.83	20.70	24.95	516.67	13.08	2.78	15.19	2.37	0.57
Godhra	89	73.00	78.00	113.33	63.77	1.15	13.30	9.95	11.48	23.75	273.32	15.36	2.24	2.34	1.78	0.405
Godhra	90	71.50	78.00	96.35	40.14	1.50	12.30	11.50	12.55	27.81	349.86	13.99	2.17	16.42	2.68	0.62
Godhra	91	73.00	78.50	98.62	41.78	1.30	12.92	12.43	13.99	23.58	328.29	14.98	2.26	19.92	2.58	0.54
Godhra	92	72.00	77.50	214.28	118.02	1.10	18.25	14.54	14.47	26.80	387.15	14.39	2.10	3.33	2.38	0.6
Godhra	93	72.00	78.50	170.30	93.18	1.15	18.82	14.06	13.42	28.91	387.89	12.95	1.48	12.20	1.74	0.425
Godhra	94	73.50	79.00	167.80	102.13	1.17	17.25	14.23	13.67	32.55	445.27	13.00	2.79	12.12	2.77	0.645
Godhra	95	68.50	74.00	168.08	57.00	1.00	18.42	12.70	13.66	36.15	493.96	14.86	2.29	8.85	2.13	0.54
Godhra	96	71	76	185.025	100.165	1.15	14.735	14.575	13.75	30.625	421.25	15.325	1.865	10.525	2.69	0.71

Table S5. Nei genetic distance matrix between studied maize lines.

Please see the excel sheet due to large sized table

Table S6. The Evanno table output from STRUCTURE HARVESTER.

K	Replications	Mean LnP(K)	St dev LnP (K)	Ln′ (K)	|Ln″(K)|	ΔK
**2**	5	-7191.00	1.20	-	-	
**3**	5	-6862.26	**1.16**	328.74	121.64	104.19
**4**	5	-6655.16	1.69	207.10	11.56	6.81
**5**	5	-6459.62	1.18	195.54	79.42	66.83
**6**	5	-6343.50	20.58	116.12	31.74	1.54
**7**	5	-6195.64	5.05	147.86	58.68	11.61
**8**	5	-6106.46	32.24	89.18	60.72	1.88
**9**	5	-5956.56	13.83	149.90	50.48	3.6
**10**	5	-5857.14	11.83	99.42	-	-

Table S7. Q table derived from STRUCTURE analysis.

Sr No.	Name of Genotype	I	II	II	Inferred sub-population	Inferred cluster through Nei's distance
**1**	I-07-14-1-2	0.149	**0.845**	0.007	II	B1
**2**	I-07-28-3-2	0.285	**0.708**	0.007	Admixture	B2
**3**	I-07-29-1-3	0.017	**0.970**	0.013	II	B1
**4**	I-07-66-1-2	0.010	**0.979**	0.011	II	B1
**5**	I-07-66-2-3	0.027	**0.965**	0.008	II	B1
**6**	I-07-66-3-2	0.030	**0.963**	0.007	II	B1
**7**	I-07-66-4-1	0.171	**0.798**	0.032	Admixture	B1
**8**	I-07-56-4-3	0.084	**0.909**	0.007	II	B1
**9**	I-077-59-5	0.089	**0.901**	0.01	II	B1
**10**	I-07-60-4-3	0.008	**0.767**	0.225	Admixture	B1
**11**	I-07-65-44-4	0.010	**0.969**	0.021	II	C
**12**	I-07-6-4-4	0.005	**0.987**	0.008	II	C
**13**	I-07-6-4-5	0.269	**0.718**	0.013	Admixture	B1
**14**	I-07-9-5	0.229	**0.742**	0.029	Admixture	B2
**15**	I-07-13-1-3	0.151	**0.844**	0.005	II	A2
**16**	IL-14-28	0.055	**0.930**	0.015	II	B1
**17**	IL-14-48	0.012	**0.878**	0.111	II	B1
**18**	IL-14-60	0.020	**0.971**	0.009	II	B1
**19**	IL-17-28	0.008	**0.969**	0.023	II	B1
**20**	IL-17-32	0.004	**0.987**	0.008	II	B1
**21**	IL-17-34	0.028	**0.966**	0.006	II	B1
**22**	IL-17-44	0.008	**0.967**	0.025	II	B1
**23**	GYL-10	0.005	**0.985**	0.01	II	B1
**24**	PFSR5-3-5	0.009	**0.983**	0.007	II	B1
**25**	IL-15-39	0.008	**0.986**	0.007	II	B1
**26**	IL-15-11	0.022	**0.851**	0.127	II	B1
**27**	IGI-1101	**0.987**	0.007	0.006	I	B1
**28**	IGI-1103	**0.947**	0.043	0.01	I	A2
**29**	GYL-2	**0.987**	0.007	0.006	I	A2
**30**	CML-307	**0.984**	0.008	0.008	I	A2
**31**	IGI-1102	**0.967**	0.012	0.02	I	A2
**32**	IGI-1104	**0.975**	0.021	0.005	I	A2
**33**	LTP-1-1	**0.986**	0.008	0.006	I	A2
**34**	LM-B-2	**0.917**	0.007	0.076	I	A2
**35**	LM-5	**0.955**	0.021	0.024	I	A2
**36**	H07R-1-3	**0.699**	0.205	0.096	Admixture	A2
**37**	GWQPM-5-3	0.274	**0.718**	0.007	Admixture	B1
**38**	GWQPM-11	0.475	**0.519**	0.006	Admixture	B2
**39**	GWQPM-17-2	0.256	**0.724**	0.02	Admixture	B2

**40**	GWQPM-22-5	0.207	**0.715**	0.079	Admixture	B1
**41**	GWQPM-26-1	0.448	**0.507**	0.045	Admixture	B2
**42**	GWQPM-26-3	0.012	**0.788**	0.200	Admixture	B2
**43**	GWQPM-40-3	0.011	**0.967**	0.023	II	B1
**44**	GWQPM-40-5	0.034	**0.935**	0.031	II	B1
**45**	GWQPM-46-2	0.112	**0.813**	0.075	II	B1
**46**	GWQPM-47-1	0.119	**0.850**	0.031	II	B2
**47**	GWQPM-55-2	0.008	**0.987**	0.005	II	B2
**48**	GWQPM-67-2	0.077	**0.885**	0.038	II	B1
**49**	GWQPM-68-4	0.055	**0.842**	0.102	II	B2
**50**	I-07-54-3-2	0.01	**0.941**	0.048	II	B1
**51**	I-07-7-3-2	**0.981**	0.015	0.004	I	A2
**52**	I-07-62-42-2	0.016	**0.949**	0.035	II	C
**53**	1820161/20-5	0.024	**0.952**	0.024	II	C
**54**	1820164/20	0.033	**0.905**	0.062	II	C
**55**	1820168/T1	0.009	**0.975**	0.016	II	C
**56**	1820213/9-20	0.005	**0.758**	0.237	Admixture	C
**57**	1820215/C1-20	0.024	**0.919**	0.057	II	C
**58**	1820228/T2	0.009	**0.925**	0.065	II	C
**59**	11820211/T2	0.015	**0.868**	0.116	II	B2
**60**	1820211/T1	**0.961**	0.019	0.019	I	B2
**61**	1820200/C1-20	**0.912**	0.008	0.08	I	A2
**62**	1820198/C1-20	**0.748**	0.026	0.225	Admixture	A2
**63**	1820197/T2	**0.985**	0.005	0.009	I	A2
**64**	1820197/T1	**0.984**	0.005	0.011	I	A2
**65**	1820167/C4-20	**0.887**	0.094	0.019	I	A2
**66**	1820194/T1	**0.962**	0.005	0.033	I	A2
**67**	1820192/4-20	**0.990**	0.003	0.007	I	A2
**68**	1820229/T2	0.403	0.009	**0.588**	Admixture	A2
**69**	I-07-33-04	**0.776**	0.014	0.210	Admixture	A2
**70**	I-07-34-3-1	0.311	0.174	**0.515**	Admixture	A1
**71**	I-07-36-1-4	0.293	0.013	**0.694**	Admixture	A1
**72**	I-07-36-2	0.162	0.013	**0.825**	III	A1
**73**	I-07-37-6-1	0.017	0.005	**0.977**	III	A1
**74**	I-07-40-4-2	0.015	0.014	**0.972**	III	A1
**75**	I-07-62-22-5	0.011	0.003	**0.985**	III	A1
**76**	1820162/T3	0.047	0.051	**0.902**	III	A1
**77**	1820166/T2	0.006	0.004	**0.989**	III	A1
**78**	1820162/T2	0.006	0.007	**0.987**	III	A1
**79**	1820162/T1	0.009	0.125	**0.867**	III	A1
**80**	1820212/T2	0.023	0.078	**0.899**	III	A1
**81**	1820214/C1-20	0.005	0.016	**0.979**	III	A1
**82**	1820228/T1	0.004	0.004	**0.992**	III	A1
**83**	1820229/T1	0.007	0.008	**0.985**	III	A1
